# β-catenin perturbations control differentiation programs in mouse embryonic stem cells

**DOI:** 10.1016/j.isci.2022.103756

**Published:** 2022-01-10

**Authors:** Elisa Pedone, Mario Failli, Gennaro Gambardella, Rossella De Cegli, Antonella La Regina, Diego di Bernardo, Lucia Marucci

**Affiliations:** 1 Department of Engineering Mathematics, University of Bristol, Bristol BS8 1UB, UK; 2 School of Cellular and Molecular Medicine, University of Bristol, Bristol BS8 1TD, UK; 3 Telethon Institute of Genetic and Medicine Via Campi Flegrei34, 80078 Pozzuoli, Italy; 4 Department of Chemical, Materials and Industrial Production Engineering, University of Naples Federico II, 80125 Naples, Italy; 5 Department of Electrical Engineering and Information Technology, University of Naples Federico II, 80125 Naples, Italy; 6 BrisSynBio, Bristol BS8 1TQ, UK

**Keywords:** Cell biology, Stem cells research

## Abstract

The Wnt/β-catenin pathway is involved in development, cancer, and embryonic stem cell (ESC) maintenance; its dual role in stem cell self-renewal and differentiation is still controversial. Here, by applying an *in vitro* system enabling inducible gene expression control, we report that moderate induction of transcriptionally active exogenous β-catenin in β-catenin null mouse ESCs promotes epiblast-like cell (EpiLC) derivation *in vitro*. Instead, in wild-type cells, moderate chemical pre-activation of the Wnt/β-catenin pathway promotes EpiLC *in vitro* derivation. Finally, we suggest that moderate β-catenin levels in β-catenin null mouse ESCs favor early stem cell commitment toward mesoderm if the exogenous protein is induced only in the “ground state” of pluripotency condition, or endoderm if the induction is maintained during the differentiation. Overall, our results confirm previous findings about the role of β-catenin in pluripotency and differentiation, while indicating a role for its doses in promoting specific differentiation programs.

## Highlights

• Moderate β-catenin levels promote EpiLCs derivation *in vitro*
• Chemical pre-activation of the Wnt pathway enhances ESC-EpiLC transition
• β-catenin overexpression tips the balance between mesoderm and endoderm
• Cell fate is influenced by the extent of β-catenin induction

## Graphical abstract

**Figure:**
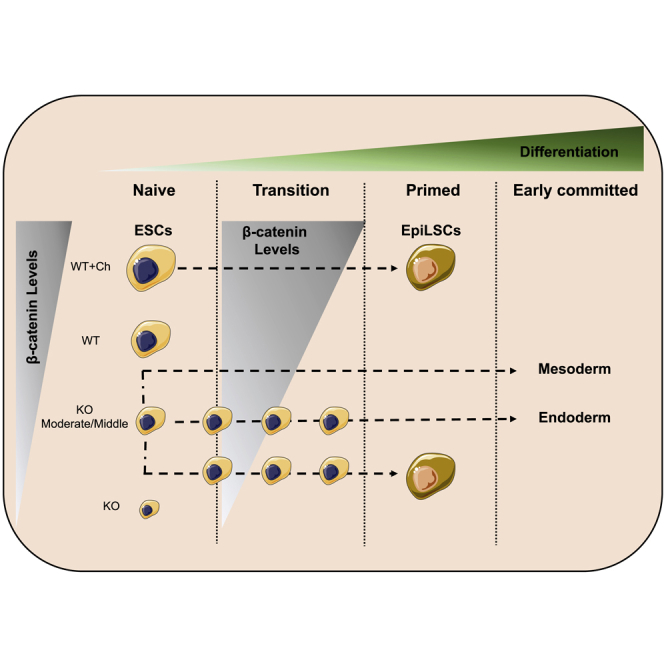

## Introduction

Pluripotent cells (PCs) are characterized by indefinite proliferative and differentiation potential and their identity is determined by the balance between signals promoting self-renewal and differentiation. The first step for stem cell differentiation is the exit from the pluripotent state, tightly controlled by signaling pathway and gene regulatory networks which can drive specific lineage commitment. During murine development *in vivo*, embryonic stem cells (hereafter called ESCs) that represent the naive pluripotent state of the early epiblast (Evans and Kaufman, 1981; Martin, 1981; Ying et al., 2008), convert into the late epiblast and finally in terminally differentiated somatic cells. ESCs can be derived from the pre-implantation epiblast; they provide an excellent system for understanding signaling pathway interplay in cell fate decision making.

In serum-based cultures, mouse ESCs are heterogeneous for the expression of pluripotency genes (Martin, 1981; Evans and Kaufman, 1981; Brook and Gardner, 1997; Williams et al., 1988; Smith et al., 1988; Ying et al., 2003a; Niwa et al., 1998; Matsuda et al., 1999; Marucci et al., 2014), while, when cultured in serum-free media supplemented with inhibitors of MEK1/2 (PD) and GSK3α/β (Chiron) and in presence or not of the leukemia inhibitory factor-LIF (2i or 2i+LIF) (Ying et al., 2008), a uniform self-renewal condition known as “ground state” of pluripotency is established; it is characterized by homogeneous gene expression (Boroviak et al., 2015; Marks et al., 2012; Godwin et al., 2017; Ghimire et al., 2018), genome demethylation (Ficz et al., 2013; Habibi et al., 2013; Leitch et al., 2013), and naive pluripotency (Nichols and Smith, 2012; Alexandrova et al., 2016). Following release from 2i or 2i+LIF, epiblast-like cells (hereafter called EpiLCs) appear *in vitro* as an intermediate of ESC differentiation (Chen et al., 2018; Hayashi et al., 2011; Buecker et al., 2014; Krishnakumar et al., 2018). EpiLCs are transcriptionally comparable to epiblast stem cells (hereafter called EpiSCs), although the latter better resemble cells of the anterior primitive steak (Kojima et al., 2014). EpiSCs, derived from the post-implantation epiblast, are capable of differentiating in all the germ layers (Brons et al., 2007; Tesar et al., 2007); they differ from ESCs in morphology, clonogenicity, gene expression, epigenome status, and, most importantly, ability to contribute to chimaeras (Ghimire et al., 2018; Tesar et al., 2007; Brons et al., 2007; Han et al., 2010). EpiSCs require Activin A and the fibroblast growth factor 2 (FGF2) (Brons et al., 2007; Tesar et al., 2007) for *in vitro* expansion; FGF signaling pathway activation, while promoting EpiSC self-renewal, induces ESC differentiation (Stavridis et al., 2007; Kunath et al., 2007; Guo et al., 2009). Different protocols based on FGF2 treatment, in combination or not with Activin A and inhibitors of the LIF/STAT3 and the Wnt/β-catenin pathways, have been proposed for the derivation and expansion of EpiLCs and EpiSCs both in serum-based and serum-free culture conditions (Joo et al., 2014; Sumi et al., 2013; Kurek et al., 2015; Hayashi et al., 2011; Gouti et al., 2014; Tsukiyama and Ohinata, 2014). Self-renewing EpiSCs have been established by simultaneous activation and inhibition of the Wnt/β-catenin pathway (Kim et al., 2013); however, the effect of these perturbations on the ESCs-EpiLCs transition has not been fully explored.

The Wnt/β-catenin is a highly conserved signaling pathway involved in ESCs self-renewal (Sato et al., 2004) and cell cycle progression (De Jaime-Soguero et al., 2017). β-catenin levels are tightly controlled by the active transcription of negative regulators working at different levels of the signaling cascade (Stamos and Weis, 2013): Axin2 (Jho et al., 2002; Chia and Costantini, 2005; Leung et al., 2002) is part of the destruction complex whereas DKK1 (Glinka et al., 1998) binds to the Wnt receptor complex attenuating cellular response upon activation of the pathway. These negative feedback loops contribute to the emergence of nonlinear dynamics in the Wnt/β-catenin pathway, proved to be important in different biological and developmental aspects (see (Pedone and Marucci, 2019) for a review), ESCs pluripotency, and somatic cell reprogramming (Marucci et al., 2014; Aulicino et al., 2014; Ho et al., 2013; Kimura et al., 2016; Lluis et al., 2008).

The role of the canonical Wnt pathway in early *in vivo* developmental stages and the requirement of its activation for ESC self-renewal have been a matter of intense research (Anton et al., 2007; Ying et al., 2008; Soncin et al., 2009; Wagner et al., 2010; Lyashenko et al., 2011; Wray et al., 2011; Faunes et al., 2013; Ye et al., 2017; Chatterjee et al., 2015; Ortmann et al., 2020; Tao et al., 2020; Theka et al., 2019; Aulicino et al., 2020). Pluripotency incompetence of β-catenin^−/−^ ECSs has been reported in two independent studies (Anton et al., 2007; Wagner et al., 2010); this phenotype was contradicted later using newly generated β-catenin^−/−^ cell lines, which showed self-renewal in both serum and 2i+LIF (hereafter called 2i/L), but presented some differentiation defects when LIF deprived (Wray et al., 2011; Lyashenko et al., 2011; Aulicino et al., 2020). Such knockout models provide an excellent *in vitro* system to study β-catenin function on ESC decision making.

Here, we take advantage of the β-catenin^−/−^ ESC line generated by Aulicino et al. (2020), where the entire β-catenin coding sequence was removed to avoid possible compensatory mechanisms from aberrant truncated isoforms, to study the effect of β-catenin perturbations on the exit from pluripotency and differentiation. Different β-catenin doses have been indirectly achieved in the past by mutating the adenomatous polyposis coli gene (APC) (Kielman et al., 2002); teratomas from the mutants with the highest β-catenin transcriptional activity showed major differentiation defects in the neuroectoderm, dorsal mesoderm, and endoderm lineages. Of note, results in Kielman et al. (2002) suggest that active β-catenin nuclear translocation (different across mutants) might also be involved in the observed differentiation impairment.

Models enabling direct modulation of β-catenin can be used to systematically associate protein perturbations to pluripotency and differentiation phenotypes. For this aim, we tuned β-catenin levels in β-catenin^−/−^ ESCs applying an improved inducible system (Pedone et al., 2019) and measured both global gene expression in ground state pluripotency (i.e., 2i/L) and following 2i/L withdrawal, as well as the efficiency of ESC-EpiLC transition *in vitro*. We demonstrated that moderate β-catenin activation in β-catenin^−/−^ ESCs (between null and wild-type levels) and moderate chemical pre-activation of the Wnt/β-catenin pathway in wild-type ESCs promote efficient EpiLC *in vitro* derivation. Finally, the transcriptome of β-catenin^−/−^ ESCs expressing different doses of exogenous β-catenin before and/or during differentiation confirmed what we and others reported about the dispensable requirement of β-catenin transcriptional activity for pluripotency establishment (Lyashenko et al., 2011; Pedone et al., 2019; Wray et al., 2011; Faunes et al., 2013; Aulicino et al., 2020), while suggesting that specific β-catenin perturbations cause a bias toward the endoderm lineage, in line with Lef-1 related results previously reported (Ye et al., 2017).

Overall, our study highlights that a synergistic effect of β-catenin doses and culture conditions controls *in vitro* ESC fate decision making at the exit from pluripotency.

## Results

### Wnt/β-catenin pathway perturbations control *in vitro* generation of EpiLC

**Figure 1:**
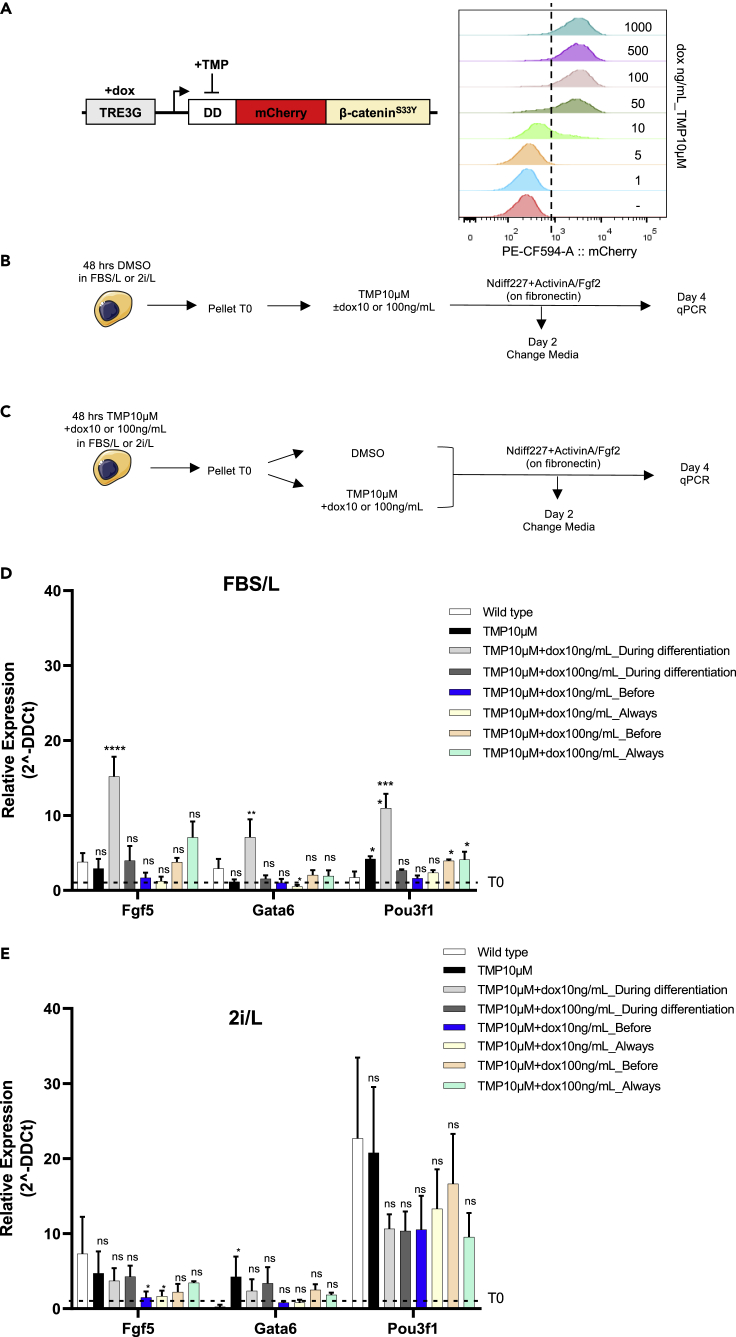
Dual-input control of β-catenin doses in EpiLC *in vitro* derivation (β-catenin^−/−^ background) A dual-input regulation system consisting of the doxycycline-responsive element and the conditionally destabilized mCherryβ-catenin^S33Y^ module. Doxycycline (dox) and trimethoprim (TMP) allow mCherryβ-catenin^S33Y^ transcription initiation and protein stabilization, respectively. (A, inset) Flow cytometry profile of C1 ESCs treated for 24 h with TMP10μM and the indicated concentrations of dox. (B and C) Experimental scheme ESC to EpiLC conversion. C1 ESCs cultured in FBS/L or 2i/L, were pre-treated either with DMSO (B) or TMP10μM and dox10–100ng/mL (C). Following 48 h of treatment, cells were seeded on fibronectin in NDiff227 and exposed to ActvinA/FGF2 and different combinations of DMSO, dox, and TMP for 4 days before being collected for RNA extraction. After 2 days, the media was changed, and the drugs were refreshed. (D and E) Fgf5, Gata6, and Pou3f1 expression in C1 ESCs cultured in FBS/L (D) or 2i/L (E) and differentiated for 4 days in NDiff227+ActivinA/FGF2 and different combination of DMSO, doxy, and TMP. Data are represented as fold change with respect to the corresponding pluripotent condition (i.e., time zero before differentiation (T0)) indicated with a dashed line. Data are means± SEM (n = 3 biological replicates). pvalues from one-way ANOVA with Bonferroni multiple comparison test computed over the wild-type ESCs are shown, ∗p<0.05, ∗∗p<0.01, ∗∗∗p<0.001, ∗∗∗∗p<0.0001

To study the role of the Wnt/β-catenin pathway in EpiLC *in vitro* derivation, we used the C1-EF1a-rtTA_TRE3G-DDmCherryβ-catenin^S33Y^ (hereafter called C1) ESC line we previously generated (Pedone et al., 2019). Briefly, β-catenin^−/−^ ESCs (Aulicino et al., 2020) were modified to stably express a doxycycline-inducible fusion protein comprising the conditional destabilizing domain (DD), the mCherry fluorescent protein, and the constitutive active β-catenin^S33Y^ (Figure 1A) (Sadot et al., 2002). The inducer molecule doxycycline (dox) enables transcriptional initiation, while trimethoprim (TMP) allows protein stabilization by inactivating the DD (Figure 1A) (Pedone et al., 2019). The use of a constitutively active and conditional β-catenin^S33Y^ form, uncoupled from upstream endogenous regulations and in a knockout background, avoids possible issues (i.e., compensatory mechanisms, genetic variation, off-target effects) resulting from endogenous protein induction or chemical pathway activation.

We confirmed in C1 cells the correct induction (Figure 1A, inset and (Pedone et al., 2019)), intracellular distribution, and functionality of the exogenous protein upon input administration (Figure S1A and (Pedone et al., 2019)). We found a dose-dependent upregulation of the β-catenin target gene Axin2 in C1 ESCs cultured with dox and TMP for 48 h, although not reaching activation comparable to Chiron 3-μM-treated wild-type cells in the case of maximum induction (Figure S1A). This result confirms our previous measurements of the total exogenous β-catenin levels induced by drugs being lower than wild-type condition, while still being active in the nucleus, thanks to the use of a mutant form, insensitive to the endogenous degradation machinery (Pedone et al., 2019).

The β-catenin^−/−^ cell line we used had already been characterized for having a transcriptional profile similar to wild-type cells in pluripotent (serum/LIF) cultures, with Wnt signaling activation repressing ESC spontaneous differentiation in dependence of β-catenin (Aulicino et al., 2020). We also previously confirmed the dispensable role of β-catenin in pluripotent culture conditions and showed, using alkaline phosphatase (AP) staining, that moderate β-catenin induction with our inducible system (using TMP10μM_dox10ng/mL) can protect cells from exiting pluripotency in the absence of both serum and LIF (Pedone et al., 2019).

Following these results, we measured the efficiency of EpiLCs derivation when different doses of exogenous β-catenin are induced in β-catenin^−/−^ cells under pluripotent conditions and/or during differentiation. To appreciate cellular response changes depending on the culture condition, C1 ESCs were expanded either in serum/LIF (hereafter called FBS/L) or in 2i/L. As prolonged culture in 2i/L results in epigenetic changes impairing normal differentiation *in vitro* and development *in vivo* (Choi et al., 2017), we opted for a short-term culture in 2i/L (3 passages).

ESCs from FBS/L or 2i/L (Figures 1B and 1C) were cultured for 48 h either in DMSO (Figure 1B) or in the presence of maximum TMP (10μM) combined with low (10ng/mL) or saturating (100ng/mL) dox (Figure 1C). The concentrations of dox were extrapolated from flow cytometry measurements of the mCherry signal to provide two doses (moderate and high) of the exogenous protein (Figure 1A, inset). To explore the effect of β-catenin perturbations on ESC-EpiLC transition, we adapted the protocol for EpiSCs culture from (Kim et al., 2013) (see STAR Methods for details), FBS/L- or 2i/L-cultured C1 ESCs were supplemented with Activin A, FGF2 (Kim et al., 2013), and different combinations of DMSO, TMP, and dox (Figures 1B, 1C and STAR Methods).

Cells were kept under these conditions for 4 days, with media refreshed after the first 2 culture days; flow cytometry showed that the mCherry florescence was only marginally influenced by the frequency media and drugs were refreshed (Figure S1B). The fluorescent reporter was expressed in a dose-dependent manner following 48h drug treatment in pluripotency conditions (Figures S1C and S1D); similarly, cells were sensitive to different concentrations of drugs and to their removal during differentiation (Figures S1E and S1F). Upon protocol completion, ESCs were analyzed for the expression of the early epiblast and epiblast-like markers Fgf5, Gata6, and Pou3f1 (i.e., Oct6) (Kalkan et al., 2017; Navarra et al., 2016; Wang et al., 2021), and of the pluripotency markers Nanog and Esrrb (Kalkan et al., 2017) by qPCR (Figures 1D, 1E, S2A, and S2B). TMP-treated (i.e., control) C1 cells did not show a significant differentiation impairment, as compared to the wild-type parental cell line (Figures 1D and 1E). We found a significant upregulation of epiblast-like genes in FBS/L-cultured C1 ESCs induced with a low amount of dox (TMP10μM_dox10ng/mL “During Differentiation” sample, Figure 1D), suggesting that this culture condition favors EpiLC conversion (Figure 1D). This effect was not observed in C1 ESCs pre-cultured in 2i/L for 3 passages (Figure 1E). As expected, upon the differentiation process, Nanog and Esrrb were efficiently downregulated (Figures S2A and S2B).

Altogether, these results indicate that both culture media and β-catenin doses strongly influence how cells respond to the ActivinA/FGF2 stimulus, with moderate β-catenin induction during the differentiation protocol facilitating the transition toward EpiLCs of FBS/L-cultured ESCs.

### Chemical activation of the canonical Wnt pathway in pluripotent conditions modulates EpiLC conversion of wild-type ESCs

The above results motivated us to explore the EpiLC conversion potential of wild-type ESCs when deprived of pluripotency factors and exposed to different chemical perturbations of the endogenous Wnt/β-catenin pathway. Simultaneous activation/inhibition of the canonical WNT pathway has been previously reported to facilitate EpiSCs derivation and *in vitro* expansion (Kim et al., 2013). However, a potential effect of such a combination was not explored in the ESC-EpiLC conversion. When activating the pathway with the Gsk3 inhibitor Chiron-99021 (Chiron), the levels of activated β-catenin will be here significantly higher than in the experiments in Figure 1, thus different results in EpiLC conversion are expected.

**Figure 2:**
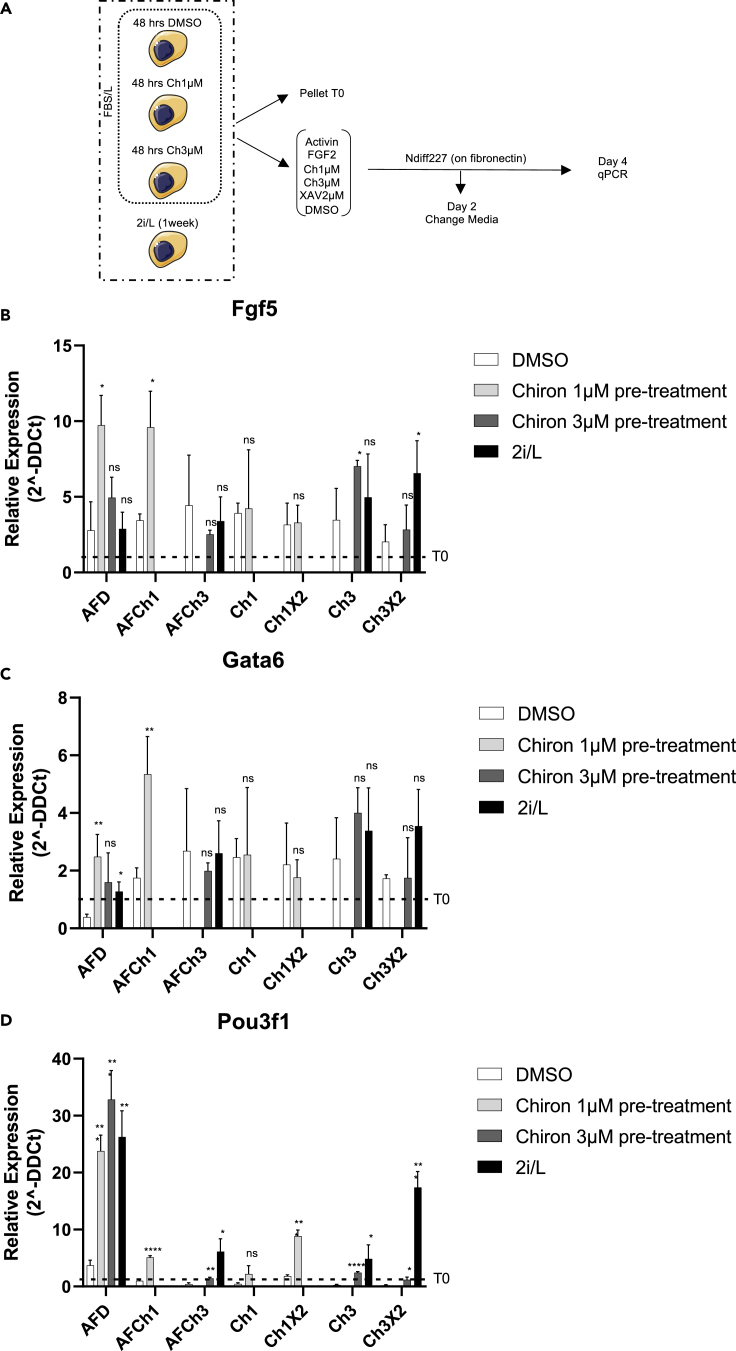
Chemical perturbation of the Wnt/β-catenin in EpiLC *in vitro* derivation (β-catenin wild-type background) (A) Experimental scheme of EpiLCs derivation. Wild-type ESCs cultured in FBS/L and pre-treated for 48 h with DMSO and Chiron (1–3 μM), or in 2i/L for 3 passages, were seeded on fibronectin in NDiff227 supplemented with different combinations of drugs (ActivinA+FGF2+DMSO (AFD); ActivinA+FGF2+Ch1μM (AFCh1); ActivinA+FGF2+Ch3μM (AFCh3); Ch1μM (Ch1); Ch1μM+XAV2μM (Ch1X2); Ch3μM (Ch3); Ch3μM+XAV2μM (Ch3X2)). After 2 days, the media was changed, and the drugs were refreshed. Expression of epiblast-like genes was measured by qPCR in pluripotent conditions (T0) and after 4 days of differentiation. (B–D) Fgf5 (B), Gata6 (C), and Pou3f1 (D) expression in DMSO, Ch1μM, Ch3μM, and 2i/L pre-cultured wild-type ESCs differentiated for 4 days in NDiff227 and the indicated combination of drugs. Data are represented as fold change with respect to the corresponding pluripotent condition (i.e., time zero before differentiation (T0)) indicated with a dashed line. Data are means± SEM (n = 3 biological replicates). pvalues from two-tailed unpaired t test computed over the DMSO are shown, ∗p<0.05, ∗∗p<0.01, ∗∗∗p<0.001, ∗∗∗∗p<0.0001

We measured the transition from ESCs to EpiLCs in a 4-day time course by qPCR (Figure 2A). Before differentiation, ESCs were cultured in FBS/L and treated for 48 h with DMSO or with Chiron to pre-activate the canonical Wnt pathway, or were maintained in 2i/L for 3 passages (i.e., 1 week; Figure 2A). At day 0, cells were exposed to different combination of drugs added to the NDiff227 (Guo et al., 2009): ActivinA+FGF2+DMSO (AFD); ActivinA+FGF2+Ch1μM (AFCh1); ActivinA+FGF2+Ch3μM (AFCh3); Ch1μM (Ch1); Ch1μM+XAV2μM (Ch1X2); Ch3μM (Ch3); Ch3μM+XAV2μM (Ch3X2) (Kim et al., 2013) (Figure 2A). The expression of the epiblast-like genes Fgf5, Gata6, and Pou3f1 and the pluripotency genes Nanog and Esrrb was analyzed by qPCR after 4 days. A change of media was performed after the first 2 culture days (Figure 2A).

In the FBS/L condition, the expression of epiblast-like genes, as compared to the standard differentiation protocol based on Activin A and FGF2 (i.e., AFD), showed mixed behaviors upon Wnt/β-catenin pathway perturbations (Figure S3A); however, pluripotency genes were significantly upregulated *vs* AFD in the majority of perturbations (Figure S3E), suggesting that the standard differentiation protocol, with no activation/inhibition of the pathway, is the most suited to support the ESC-EpiLC transition in FBS/L.

Next, we differentiated cells after pre-activation of the Wnt/β-catenin pathway in FBS/L condition (Figures S3B, S3C, S3F, and S4A) or 1 week of pre-culture in 2i/L (Figures S3D and S4B). Upon Chiron 1 μM, but not Chiron 3 μM, pre-treatment in FBS/L, the expression of EpiLC genes with the AFD protocol was higher as compared to the DMSO condition (Figures 2B–2D and S3A–S3C). In Chiron 1μM pre-treated and AFD-differentiated ESCs, the downregulation of pluripotency genes was comparable to that observed in 2i/L cultured cells, which can efficiently differentiate (Figures S4C and S4D (Hackett et al., 2018; Hayashi et al., 2011)). Instead, pluripotency genes were not efficiently downregulated when the Wnt/β-catenin pathway was perturbed also during differentiation in ESCs pre-activated with Chiron 1–3 μM in FBS/L (AFCh1 and Ch1 in Figures S3F and S4D; AFCh3 and Ch3 in Figures S4A and S4C).

Altogether, these results indicate that intermediate chemical pre-activation of the Wnt pathway in FBS/L-cultured wild-type cells favors the ESC-EpiLC transition using the standard differentiation protocol (AFD).

### Transcriptome and WGCNA analysis of ESC exit from pluripotency with varying β-catenin doses

Next, we studied C1 ESCs exit from the “ground state” of pluripotency, i.e., upon 2i/L withdrawal, by RNA sequencing; such monolayer differentiation protocol does not induce a specific cell fate and is well suited to observe possible β-catenin-dependent differentiation bias (Kalkan et al., 2017).

**Figure 3:**
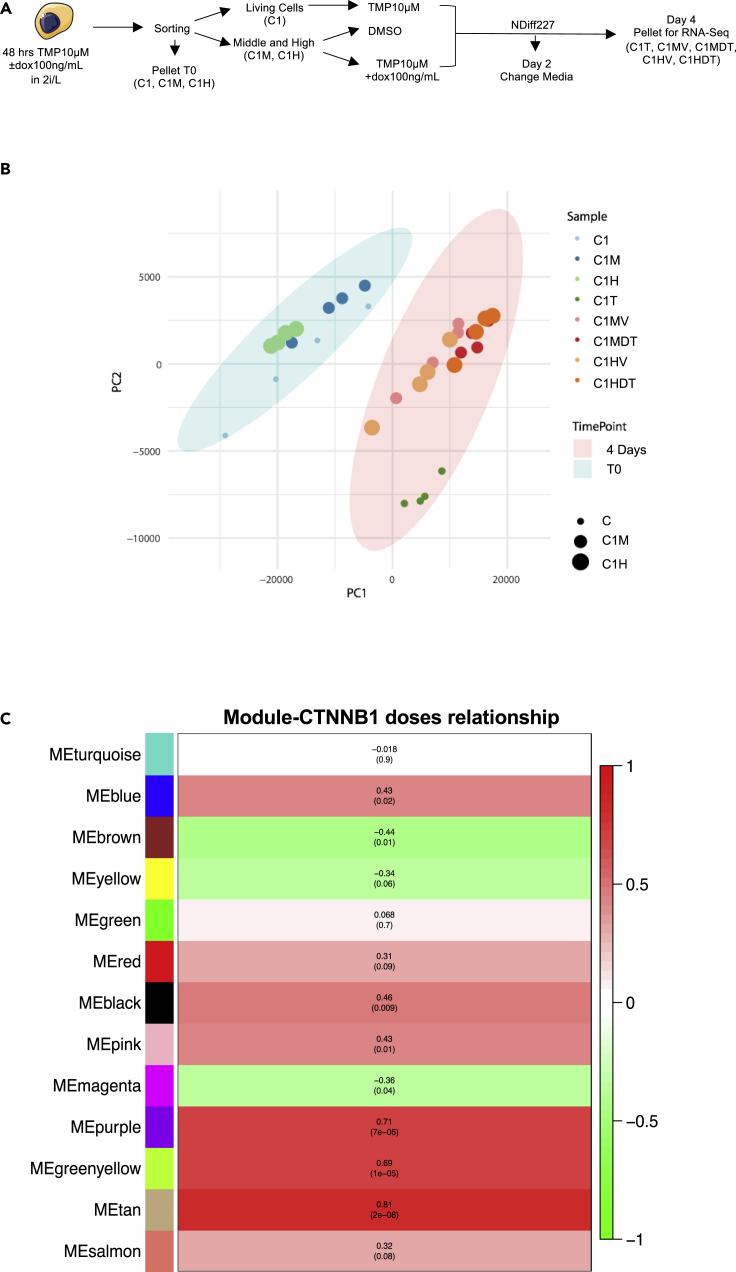
Transcriptome analysis of monolayer differentiation experiments upon β-catenin perturbations and WGCNA of the genes correlating with β-catenin doses (A) Experimental scheme of the monolayer differentiation protocol. 2i/L C1 ESCs were pre-treated with TMP10μM and dox100ng/mL for 48 h; living cells were then sorted from the Dapi negative fraction of TMP-treated cells (C1), whereas β-catenin-induced cells from dox/TMP-treated samples were FACS-sorted from the mCherry fraction and divided into middle (C1M) and high (C1H) subpopulations. 1.5×10^4^ cells/cm^2^ from each individual population were then seeded on gelatin in NDiff227 supplemented with DMSO or TMP±dox100ng/mL. After 4 days of differentiation in NDiff227, cells were collected and processed for the RNA sequencing. During the protocol, the media was changed, and the drugs were refreshed after 2 culture days. (B) Principal component analysis (PCA) of all samples following batch correction with Combat-seq method; the average of replica is shown. (C) Eigen modules correlating with β-catenin doses; the Pearson Correlation Coefficient (*r*) and relative pvalue are shown

C1 ESCs cultured in 2i/L for 3 passages (i.e., 1 week) were treated for 48 h with saturating concentrations of dox and TMP (100ng/mL and 10 μM, respectively) to induce the expression of the exogenous fusion protein (Figure 3A). Taking advantage of the mCherry-tag for exogenous β-catenin induction, dox/TMP-treated C1 ESCs were sorted into two different subpopulations: C1 with middle and high β-catenin levels (hereafter called C1M and C1H samples, respectively; Figures 3A and S5A). We also checked activation of the pathway in sorted cells by measuring Axin2 via qPCR (Figure S5B) and found a dose-dependent activation of this target gene. Sorted cells were cultured in NDiff227 media± inducers for 4 days before being transcriptionally profiled (see STAR Methods for details; Figure 3A). Sequencing informed about the transcriptome of pluripotent C1, C1M, and C1H ESCs, and of their differentiated counterparts (Day 4 samples) cultured in NDiff227 and DMSO (i.e., upon dox/TMP withdrawal; hereafter called C1MV and C1HV), or in NDiff227 and TMP/dox (hereafter called C1MDT and C1HDT) during differentiation. The C1 and C1T samples refer to cells treated only with TMP10μM in the pluripotent and differentiated states, respectively. We measured mCherry levels upon addition/removal of dox and TMP and confirmed dose-/administration time-dependent upregulation of exogenous β-catenin (C1MDT and C1HDT; Figure S5C).

To investigate the extent of the batch effect, we applied the ComBat-Seq method (Zhang et al., 2020) considering sample replicates as different batches and preserving differences among sample types. ComBat-Seq applies a set of statistical corrections to remove the batch effect in the dataset and thus reduce spurious correlations between genes. We then compared, for each sample, the 2D correlation value between the normalized expression profiles with and without using the ComBat-Seq method (Figure S5D). It can be appreciated that the samples are very similar (i.e., correlations close to 1 for each sample) before and after the batch correction thus suggesting that ComBat-Seq applies a negligible correction. Indeed, the principal component analysis (PCA) after batch correction (Figure 3B) does not substantially differ from the one in Figure S6A (no correction).

Principal components analysis (PCA, Figure 3B) showed three main clusters: one group included C1, C1M, and C1H in the “ground state” of pluripotency, another group included differentiated C1T ESCs and the final group contained all perturbed samples (C1MV, C1HV, C1MDT, and C1HDT) after 4 days of differentiation.

Next, to explore the biological processes associated with β-catenin perturbations, we used weighted gene correlation network analysis (WGCNA) (Langfelder and Horvath, 2008), a gene network approach that starting form transcriptional data allows us to identify highly co-expressed group of genes (a.k.a. modules) and associate them to the phenotypes or experimental conditions under investigation. By applying WGCNA on our transcriptional data, we identified 13 unique gene modules (Figure S6B; see STAR Methods for details), of which seven (i.e., Green, Blue, Black, Brown, Turquoise, Yellow, and Pink; Figure S6C and Table S1) were significantly correlated with differentiation time, thus containing genes playing a key role during differentiation (Figure S6C and STAR Methods). In particular, while four out of the seven gene modules (i.e., Green, Blue, Black, and Brown) were positively correlated with differentiation time and thus highly co-expressed (i.e., active) after 4 days of differentiation, three modules (i.e., Turquoise, Yellow, and Pink) resulted instead anti-correlated with differentiation time, meaning that those genes are highly co-expressed at T0 but not after 4 days of differentiation. Gene Ontology Enrichment Analysis (GOEA) of the most representative genes (Table S1 and STAR Methods) from the modules highly co-expressed at T0 showed a significant enrichment (FDR <0.05) in biological processes related to regulation of tissue remodeling, embryonic and forebrain development, stem cell population maintenance, cell homeostasis (Figure S7A and Table S1, Turquoise), nuclear division, meiosis and organelle fission (Figure S7B and Table S1, Pink). On the other hand, gene modules positively correlated with differentiation time (i.e., genes active at day 4) showed a significant enrichment (FDR <0.05) in biological processes related to translation, rRNA processing, ribosomal biogenesis (Figures S7C, S7E and Table S1, Green and Brown), protein transport, processes associated with cellular respiration (Figure S7D and Table S1, Blue), positive regulation of growth, ncRNA processing, and neuronal tube formation and development (Figure S7E and Table S1, Brown). Of note, there were no significantly enriched BPs for the genes from the Black, Yellow, and Pink modules (Table S1).

Next, to gain more information about the effects of β-catenin at the exit from pluripotency, we also correlated each module with β-catenin doses (basal, moderate, and high, Figure 3C) and found three gene modules gradually increasing their co-expression with increasing β-catenin concentration (i.e., Tan, Purple, and Green/Yellow; Figure 3C and Table S1). The biological processes (Table S1 and STAR Methods) corresponding to these modules showed enrichment in cell division (Figure S8A and Table S1, Tan), metabolism, and negative regulation of neuronal death (Figure S8B and Table S1, Purple).

Overall, the WGCNA analysis confirmed the expected major transcriptional and metabolic changes associated with the exit from the pluripotent status and confirmed previously reported β-catenin functions on cell survival and proliferation (De Jaime-Soguero et al., 2017).

Next, we performed differential gene expression (DGE) analysis between specific pairs of samples and performed GOEA to identify the involved biological processes (red bars in Figures S9A–S9D, S10A, and S10B). Starting from the pluripotent condition, when comparing C1M and C1H with C1, we found that the first 10 biological processes with an FDR <0.05 were mainly related to cell cycle and metabolism (Figures S9A and S9B; Tables S2 and S3). Interestingly, the genes exclusively upregulated in C1H were related to tissue differentiation (i.e., eye morphogenesis and urogenital system development) and DNA methylation involved in gamete generation (Figure S9B; Table S3). Only a few signaling pathways were enriched in C1M ESCs compared to the control cell line C1 (Tables S2 and S3). These results, together with the PCA in Figure 3B, confirm previous observations about the dispensable function β-catenin has in pluripotent culture conditions (Lyashenko et al., 2011; Pedone et al., 2019; Wray et al., 2011; Aulicino et al., 2020) and suggest a bias toward differentiation in C1H ESCs (Figure S9B; Table S3).

**Figure 4:**
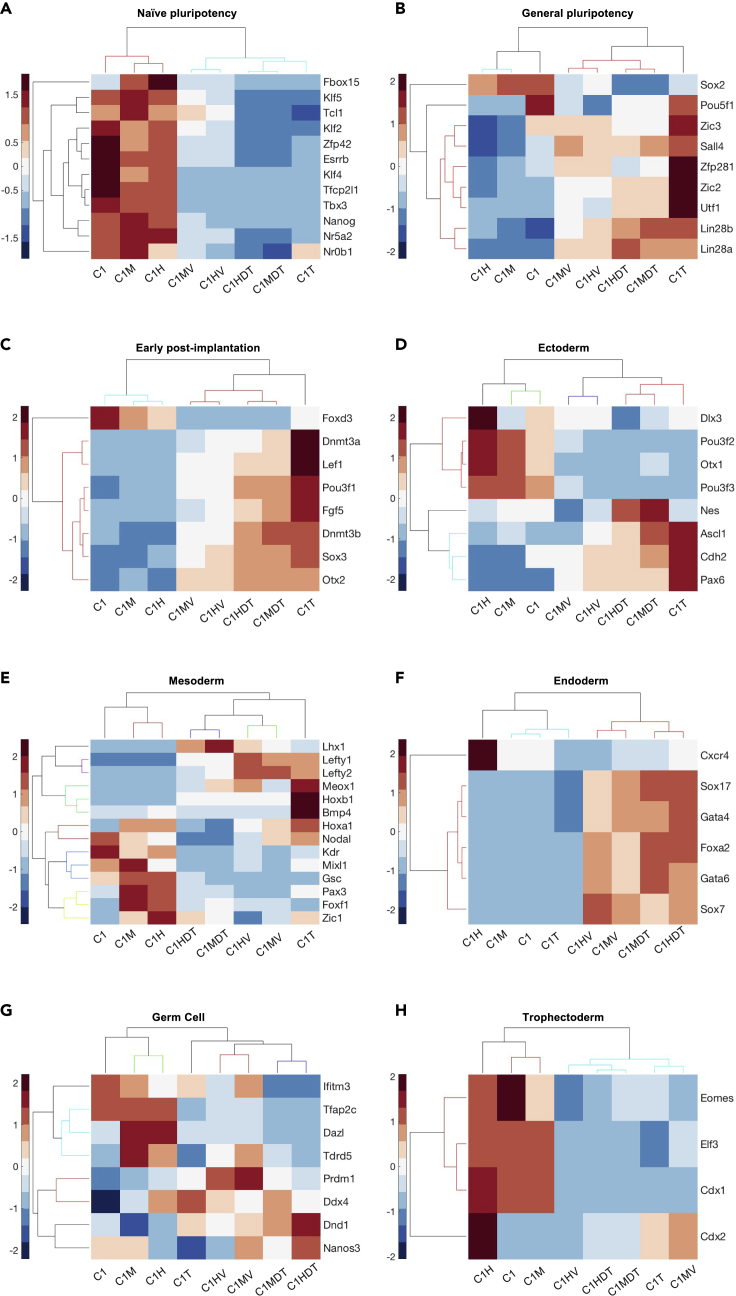
Gene ontology and clustergram of the differential expressed genes in control and perturbed ESCs (A–H) Clustergram over heatmaps of naive (A) and general pluripotency (B), early post-implantation (C), ectoderm (D), mesoderm (E), endoderm (F), germ cell (G), and trophectoderm (H) lineages from pluripotent and differentiated ESCs expressing different β-catenin amount. Each column is the average of 4 samples from the same experiment.

We then analyzed the genes differentially expressed at day 4 upon β-catenin perturbation as compared to C1T ESCs. The upregulated genes gave the major contribution to the significantly enriched BPs (Figures S9C, S9D, S10A, S10B; Table S4. Differentially expressed genes, C1MV vs C1T, related to Figure 4, Table S5. Differentially expressed genes, C1MDT vs C1T, related to Figure 4, Table S6. Differentially expressed genes, C1HV vs C1T, related to Figure 4, Table S7. Differentially expressed genes, C1HDT vs C1T, related to Figure 4), while the downregulated genes only contributed to enrich a few processes, namely general metabolic processes (e.g., regulation of transporter and cation channel activity) and mesenchymal to epithelial transition (Figures S9C, S9D, S10A, S10B; Table S4. Differentially expressed genes, C1MV vs C1T, related to Figure 4, Table S5. Differentially expressed genes, C1MDT vs C1T, related to Figure 4, Table S6. Differentially expressed genes, C1HV vs C1T, related to Figure 4, Table S7. Differentially expressed genes, C1HDT vs C1T, related to Figure 4). Genes exclusively upregulated in the C1MV *vs* C1T comparison belonged to the mesoderm lineage (i.e., cardiovascular system development; Figure S9C and Table S4), while, in the C1MDT *vs* C1T comparison, upregulated genes were enriched for the endoderm lineage (i.e., urogenital system; Figure S9D and Table S5). Nevertheless, mesoderm and endoderm lineages were represented in both comparisons. GO performed on the C1HV and C1HDT comparisons with C1T showed only a few differences in the enriched BPs, that indeed did not define a bias toward a specific lineage (Figures S10A and S10B; Tables S6 and S7).

These results suggest that the major changes in the differentiation program initiated upon 2i/L withdrawal are induced by moderate β-catenin doses and are influenced by the timing of protein induction. The pathway enrichment analysis showed the upregulation of protein metabolism in C1MV and C1HV (Tables S4 and S6), MAPK signaling pathway (Table S4) in C1MV, and ECM-receptor interaction and PI3K-AKT signaling pathway in C1HV (Table S6).

To gauge insights into specific differentiation programs, we selected sets of markers for naive and general pluripotency, early post-implantation epiblast, ectoderm, mesoderm, endoderm, germ cell, and trophectoderm (Kalkan et al., 2017), and clustered our samples according to their expression.

Naive pluripotency genes were downregulated upon differentiation in all samples, indicating the successful exit of cells from pluripotency (Figure 4A). Pluripotent C1M and C1H samples clustered together (Figure 4A) although close to C1 ESCs confirming that β-catenin is dispensable for pluripotency maintenance. ESCs differentiated in presence of DMSO (i.e., C1MV and C1HV; Figure 4A) clustered together, similarly to samples differentiated in presence of dox and TMP (i.e., C1MDT and C1HDT; Figure 4A); still, a large number of genes (e.g., Klf5, Tcl1, Klf2 and Nr0b1) showed a different pattern among differentiated samples C1T, C1MV, and C1HV, discriminating ESCs with different β-catenin doses (Figure 4A). These results support the hypothesis of a β-catenin-dependent effect on transcriptional changes.

A similar clustering across pluripotent samples was observed for general pluripotency markers (Figure 4B). In the majority of differentiated samples, Sox2 was downregulated while Utf1, Zfp281, and Lin28 were upregulated (Figure 4B), in accordance with previous reports (Zhang et al., 2016; Fidalgo et al., 2016; Luo et al., 2015; Betschinger et al., 2013). Under differentiated culture condition, Zfp281, Zic2, and Utf1 were downregulated in β-catenin-induced cells (i.e., C1MV, C1HV, C1MDT, and C1HDT) as compared to C1T ESCs (Figure 4B). Zfp281 is a Zinc finger transcription factor implicated in pluripotency (Brandenberger et al., 2004; Wang et al., 2008), and recently reported as a bidirectional regulator of the ESC-EpiSC transition in cooperation with Zic2, another zinc finger protein (Mayer et al., 2020). The undifferentiated embryonic cell transcription factor 1 (Utf1) is expressed in ESCs and plays an important role in the exit from pluripotency (Jia et al., 2012; Kooistra et al., 2009). The concomitant reduction of Zfp281, Zic2, and Urf1 in the comparison between C1T with both DMSO- and dox/TMP-treated samples suggests a global change in the chromatin organization of β-catenin-induced ESCs en route to differentiation (Figure 4B). Finally, almost all the genes from this panel showed different behaviors in DMSO- (i.e., C1MV and C1HV; Figure 4B) *vs* dox/TMP-treated samples (i.e., C1MDT, C1HDT; Figure 4B), confirming that the extent of β-catenin induction affects cell identity.

Early post-implantation epiblast genes were mostly upregulated in primed ESCs compared to the pluripotent condition, with no evident differences across treatments in naive ESCs (Figure 4C). The exception was Foxd3, which was downregulated in both naive and primed β-catenin-induced cells as compared to the controls C1 and C1T ESCs (Figure 4C). Interestingly, Dnmt3a and Dnmt3b showed a reduction in C1MV/C1HV and C1MDT/C1HDT samples as compared to the control C1T (Figure 4C); also, samples constantly exposed to dox/TMP (i.e., C1MDT and C1HDT) showed higher Dnmt3a expression than DMSO-treated ESCs (i.e., C1MV and C1HV; Figure 4C). Dnmt3a, b, and Foxd3 are DNA and chromatin remodeling factors, respectively; Dnmt enzymes methylate genomic regions, whereas Foxd3 reduces active and enhances inactive histone marks by recruiting the lysine-specific demethylase 1 (Lsd1) (Respuela et al., 2016). The reduced expression of those genes in β-catenin-induced cells, including the pluripotent markers Utf1 discussed above, suggests that cells exposed to time/dose varying β-catenin levels present a differentially methylated DNA status during the exit from pluripotency.

We then screened for a large panel of lineage-priming factors. Ectoderm lineage markers showed a dose-dependent upregulation of related genes in pluripotent cells (compare C1, C1M, and C1H; Figure 4D); following 2i/L withdrawal, the clustering resembled those of previous sets (Figures 4A–4C), with samples grouping for the duration of treatment (i.e., C1MV/C1HV and C1MDT/C1HDT grouping together; Figure 4D). Genes from this lineage had different expression across samples, making difficult to identify a clear pattern associated with β-catenin perturbations.

When looking at mesoderm markers (Figure 4E), differentiated samples clustered similarly to the previous dataset. The first group of genes (i.e., Lhx1, Lefty1/2, Meox1, Hoxb1, and Bmp4) was mainly upregulated upon differentiation, whereas the second group (i.e., Nodal, Kdr, Mixl1, Gsc, Foxf1, and Zic1) got downregulated when exiting from pluripotency (Figure 4E). Although the pattern of individual genes was hard to interpret, we observed the behavior of C1T ESCs was very different from all differentiated β-catenin-induced samples, stressing the relevance of β-catenin for mesoderm specification (Lyashenko et al., 2011) and suggesting that its induction is diminishing mesoderm commitment.

The endoderm lineage was the most influenced by β-catenin perturbations: C1T cells were unable to induce the expression of endoderm-related genes (compare C1 and C1T; Figure 4F), whereas in all perturbed ESCs their expression increased over time. As previously observed (Figures 4A–4E), samples clustered together based on the duration of β-catenin induction rather than on the gene dose (i.e., C1MV/C1HV and C1MDT/C1HDT; Figure 4F). C1HDT cells showed the highest expression for the 50% of the endoderm-associated genes (namely, Cxcr4, Gata4 and Sox7) as compared to all other differentiated samples (i.e., C1T, C1MV, C1MDT, and C1HV). These observations support previous knowledge about the β-catenin requirement for endoderm organization (Engert et al., 2013; Lyashenko et al., 2011).

In the analysis of the germ cell lineage markers, all genes showed a rather heterogeneous expression pattern across samples (Figure 4G). Pluripotent C1M and C1H clustered together and close to C1, and differentiated samples clustered based on the duration of β-catenin perturbation (i.e., C1MV/C1HV and C1MDT/C1HDT; Figure 4G).

Finally, when looking at trophectoderm markers (Figure 4H), clustering showed similarity of C1 and C1M, as for the ectoderm and endoderm lineages (Figures 4H and 4J, respectively). Of note, 90% of trophectoderm genes, with the exception of Cdx2, got downregulated during differentiation in all the conditions (Figure 4H). Eomes was recently reported to control the exit from pluripotency by acting on the chromatin status (Tosic et al., 2019); its behavior in naive C1M and C1H ESCs suggests a different chromatin conformation in pluripotent cells induced for β-catenin (Figure 4H).

Accounting for the fact that ectoderm is a default linage of the monolayer differentiation protocol we applied (Ying et al., 2003b), overall our sequencing results suggest that β-catenin induction in a knockout background favors rescuing defects in differentiation toward endoderm more than mesoderm. Indeed, mesodermal genes were mostly downregulated when β-catenin was induced, whereas endodermal genes were all upregulated as compared to the control (Figures 4E and 4F). Moreover, we observed that lineage differentiation was influenced by the duration of protein induction rather than by its dose. Accordingly, there was a transition from mesoderm to endoderm following moderate but continuous β-catenin induction (compare C1MV and C1MDT in Figures S9C and S9D). Nevertheless, endoderm was an enriched gene ontology in all considered comparisons (Figures S9C, S9D, S10A, and S10B). Finally, the observed expression of pluripotency markers Zfp281, Zic2, and Utf1, the early post-implantation markers Dnmt3a-b and Foxd3 and the trophectoderm marker Eomes suggest a reorganization of the epigenome in naive C1M and C1H ESCs and upon monolayer differentiation of C1MV, C1MDT, C1HV, and C1HDT ESCs.

## Discussion

The role of the Wnt/β-catenin pathway as a pluripotency gatekeeper has been matter of many studies and debates (Sato et al., 2004; Ogawa et al., 2006; Hao et al., 2006; Singla et al., 2006; Anton et al., 2007; Takao et al., 2007; Kielman et al., 2002); while modulation of the canonical Wnt pathway has been extensively proved to be important for EpiSC *in vivo* derivation (Tsukiyama and Ohinata, 2014; Sugimoto et al., 2015), self-renewal (Sumi et al., 2013), and *in vitro* lineage differentiation (Liu et al., 2017; Osteil et al., 2019; Kurek et al., 2015), the relevance of β-catenin doses for the exit from pluripotency and for ESCs-EpiLCs direct transition has not been explored thoroughly.

In this work, we found that genetic β-catenin manipulation or chemical perturbation of the canonical Wnt pathway control ESC fate at the exit from pluripotency, providing new insights into the role of specific doses while confirming previous finding about the transcriptional role of β-catenin in pluripotency and early differentiation.

Using two different cellular models, we found that, upon FBS/L cultures, moderate β-catenin induction in differentiating β-catenin^−/−^ ESCs or moderate pre-activation of the Wnt/β-catenin pathway in pluripotent wild-type ESCs increase the efficiency of the ESCs-EpiLCs conversion. Pharmacological activation of the Wnt/β-catenin pathway in wild-type ESCs gave different results as compared to genetic β-catenin induction in β-catenin^−/−^ ESCs (Figures 1,2, and S1–S4). This observation could be explained by β-catenin-induced genetic variation reported in Ortmann et al. (2020). Ortmann et al. demonstrated that β-catenin fluctuations in naive pluripotent stem cells from different genetic backgrounds strongly influence how efficiently cells will differentiate (Ortmann et al., 2020). We believe that, by using β-catenin^−/−^ cells and inducing a β-catenin form which is insensitive to endogenous regulations, we abolished physiologic fluctuations and therefore mitigated the effect of genetic variation on cell differentiation.

Simultaneous activation and inhibition of the Wnt/β-catenin pathway has been previously reported to maintain EpiSCs self-renewal (Kim et al., 2013): Kim et al. demonstrated that EpiSCs can be maintained in Chiron3μM/XAV2μM cultures with self-renewal regulated by both Axin2 and β-catenin. Our results suggest that the observation reported for EpiSCs (Kim et al., 2013) could also stand for the EpiLC derivation.

Overall, we confirmed the effect β-catenin has on preparing cells to appropriately respond to the differentiation stimuli previously reported (Ortmann et al., 2020), suggesting that both the duration and the dose of β-catenin induction control cell differentiation *in vitro*.

RNA sequencing performed in ESCs at the exit from the naive “ground state” of pluripotency (Kalkan et al., 2017) showed that, in β-catenin-expressing cells (in particular C1MV), Dnmt3a and Dnmt3b had an expression pattern similar to the one observed in Rex1-high ESCs differentiated using a similar protocol (Kalkan et al., 2017), indicating that moderate β-catenin induction in naive ESCs influences DNA methylation associated with the exit from pluripotency. β-catenin-dependent changes in DNA methylation have been previously reported in ESCs cultured for several passages in FBS/L (Theka et al., 2019). Theka et al. concluded that constant activation of the Wnt/β-catenin is necessary to guarantee adequate DNA methylation profiles. We also observed that persistent β-catenin induction (i.e., before and during differentiation, C1MDT and C1HDT; Figure 4C) partially restores the expression of Dnmt3a/b, which got downregulated following transient β-catenin induction (i.e., before differentiation, C1MV and C1HV; Figure 4C). Of note, we pre-cultured ESCs in 2i/L for 3 passages (1 week), a condition that strongly influences DNA methylation (Ficz et al., 2013; Habibi et al., 2013; Leitch et al., 2013). It would be interesting to assess the methylation state of specific genomic region, including imprinting control regions (ICRs), in response to dose- and time-varying β-catenin perturbations.

We observed a significant upregulation of endodermal genes in β-catenin induced cells, indicating a requirement of β-catenin for this specific fate. This phenotype was previously reported in the β-catenin null cell line generated by Lyashenko et al. (Lyashenko et al., 2011), where the defect in endoderm lineage differentiation was rescued by overexpressing both wild-type or transcriptional incompetent β-catenin; in contrast, mesoderm and ectoderm induction seemed to not require β-catenin (Lyashenko et al., 2011). With our approach that enables dose- and time-controlled β-catenin induction, we also suggest that the ectoderm lineage is not affected by β-catenin loss. Different results were reported in Tao et al. (2020), where β-catenin knockdown increased neural differentiation (Tao et al., 2020); given differences in the approaches used for β-catenin perturbations and culture conditions, additional studies (possibly also in other cell lines, and at the single-cell level) would be required for a direct comparison.

In the future, it will be of great interest to use our inducible system to interrogate the effect of a wider range of β-catenin doses and, possibly, temporal dynamics on stem cell identity and to further investigate the role of the β-catenin transcriptional activity in pluripotent and differentiated cells of both murine and human origin (La Regina et al., 2021).

### Limitations of the study

We acknowledge the present study did not characterize the effect of high (i.e., above the wild-type levels) and/or dynamic β-catenin levels on cell decision making. Moreover, we did not consider comparing transcriptionally competent *vs* incompetent exogenous β-catenin; further studies uncoupling those two functions would be required and useful to fully unveil β-catenin-driven stem cell identity.

## STAR★Methods

### Key resources table

**Table:** 

REAGENT or RESOURCE	SOURCE	IDENTIFIER
**Chemicals, peptides, and recombinant proteins**		

Dulbecco’s modified Eagle’s medium (DMEM)	Sigma	Cat# D5796-6X500ML
Phosphate-buffered saline (PBS)	Sigma	Cat# D8537-6X500ML
Fetal Bovine Serum (FBS)	Sigma	Cat# F7524-500ML
GlutaMAX (100X)	Gibco	Cat# 35050-038
2-Mercaptoethanol 50mM	Gibco	Cat# 31350-010
Sodium Pyruvate 100mM (100X)	Gibco	Cat# 11360-039
MEM NEAA (100X)	Gibco	Cat# 11140-035
Penicillin-Streptomycin	Sigma	Cat# P4458-100ML
Murine LIF	Peprotech	Cat# 250-02-25UG
NDiff 227	Takara	Cat# Y40002
Chiron-99021	Selleck	Cat# S1263
PD0325901	Selleck	Cat# S1036
Doxycycline (dox)	Sigma	Cat# D9891
Trimethoprim (TMP)	Sigma	Cat# T7883
Human ActivinA	Peprotech	Cat# 120-14E
Human FGF2	Peprotech	Cat# 100-18B
XAV939	Sigma	Cat# 575545
4′,6-diamidino-2-phenylindole (DAPI)	Sigma	Cat# D9542
PureLink RNA Mini Kit	Invitrogen	Cat# 10307963
RevertAid Reverse Transcriptase	Thermo Fischer	Cat# EP0441
RiboLock RNase Inhibitor	Thermo Fischer	Cat# EO0384
dNTP Mix	Thermo Fischer	Cat# R0191
Random Hexamers Primers	Thermo Fischer	Cat# SO142
iTaq Universal SYBR Green Supermix	Bio-Rad	Cat# 1725120
RNeasy Plus Mini Kit	Qiagen	Cat# 74134

**Deposited data**		

Sequencing Raw and analyzed data	This paper	GEO: GSE148879

**Experimental models: cell lines**		

Wildtype Embryonic Stem Cells	Pedone et al. (2019)	N/A
C1-EF1a-rtTA_TRE3G-DDmCherryβ-catenin^S33Y^ (C1)	Pedone et al. (2019)	N/A

**Oligonucleotides**		

See Table S8 for a list of oligonucleotides		

**Software and algorithms**		

QuantSeq 3′ mRNA-Seq Library Prep Kit FWD	illumina	Cat# 015.96
bcl2fastq (version v2.20.0.422)	Illumina	http://emea.support.illumina.com/content/dam/illuminasupport/documents/documentation/software_documentation/bcl2fastq/bcl2fastq2-v2-20-software-guide-15051736-03.pdf
bbduk software (bbmap suite 37.31)	Joint Genome Institute (JGI)	https://jgi.doe.gov/data-and-tools/bbtools/bb-tools-user-guide/usage-guide/ (bbmap suite 37.31)
STAR 2.6.0a3	Dobin et al. (2013)	N/A
Weighted Gene Correlation Network Analysis (WGCNA) package	Langfelder and Horvath (2008)	N/A
Dynamic tree cut algorithm from dynamicTreeCut package	Langfelder et al. (2008)	N/A
ClusterProfiler package	Yu et al. (2012)	N/A
Clustergram	MathworksMatlab R2019a, update 9.6.0.1307630	https://www.mathworks.com/help/bioinfo/ref/clustergram.html

### Resource availability

#### Lead contact

Further information and requests can be addressed to Lucia Marucci (lucia.marucci@bristol.ac.uk).

#### Materials availability

This study did not generate new unique reagents.

### Experimental model and subject details

#### Cell line derivation

C1 cell lines were previously derived in Pedone et al. (2019) by a double lentiviral infection of β-catenin^−/−^ ESCs (Aulicino et al., 2020) with the EF1a-rtTA (Neomycin) plasmid followed by the pLVX_TrE3G-DDmCherryβ-catenin^S33Y^(Puromycin). Cells were selected with Neomycin after the first round and with Puromycin after the last infection.

ESCs were cultured on gelatin-coated dishes in Dulbecco’s modified Eagle’s medium (DMEM) supplemented with 15% fetal bovine serum (FBS), 1x nonessential amino acids, 1x GlutaMax, 1 x 2-mercaptoethanol, 1x Penicillin-Streptomycin and 1000 U/mL LIF. To note, for the 2i/L culture, cells were kept for 3 passages (around 1 week) in serum-free NDiff227-based media supplemented with 1000 U/mL LIF, 3μM of the GSK-3α/β inhibitor Chiron-99021 and 1μM of the MEK inhibitor PD0325901.

### Method details

#### Epiblast-like cell (EpiLC) derivation

For EpiLC derivation *in vitro* we adapted the protocol for EpiSCs culture reported in Kim et al. (2013), except that we employed N2B27 medium (Ying and Smith, 2003; Guo et al., 2009). Briefly, ESCs cultured in FBS/L or pre-cultured in 2i/L for 3 passages were seeded at the confluence of 1.5×10^4^ cells/cm^2^, on 10 μg/mL Fibronectin-coated 12-well plates in NDiff227. According to the experiment in Figures 1D, 1E, S2A, and S2B, cells were stimulated with DMSO, TMP10μM, dox10–100ng/mL, human ActivinA 10ng/mL and human FGF2 10ng/mL, whereas in Figures 2, S3, and S4, cells were exposed to ActivinA 10ng/mL human FGF2 10ng/mL, Chiron1-3μM and the XAV939 2μM. Treatments were performed during 4 days with the media and drugs refreshed after the first 2 culture days. The concentration of ActivinA, human FGF2 and XAV939 were the same used in Kim et al. (2013).

#### Monolayer differentiation

2i/L pre-cultured C1 ESCs were sorted based on β-catenin levels. Control C1, Middle (C1M) and High (C1H) expressing ESCs were plated at 1.5×10^4^ cells/cm^2^ on gelatin-coated 12-well plates in plain NDiff227 and stimulated with DMSO or TMP10μM±dox10–100ng/mL for 4 days with the media and drugs refreshed after the first 2 culture days (Figure 3A).

#### Drugs pre-treatment

Some experimental conditions required pre-treatment of cells. For β-catenin induction in Figures 1C–1E, S2A, and S2B, C1 ESCs cultured in FBS/L or 2i/L were stimulated for 48 h with TMP10μM and dox10–100ng/mL before EpiSC differentiation, whereas for pre-activation of the canonical Wnt pathway in Figures 2, S3 and S4, wild type ESCs were exposed for 48 h to Chiron1-3μM (Figures 2A–2D, S3B, S3C, S3F, S4A, S4C, and S4D) or cultured for 3 passages in 2i/L (Figures 2A–2D, S3D and S4B–S4D), before the differentiation. To note, all experiments were performed with ESCs under FBS/L or 2i/L culture conditions as indicated in figures. RNA-seq transcriptional profiling was performed only with 2i/L pre-cultured ESCs.

#### Flow activated cell sorting (FACS)

2i/L pre-cultured ESCs were washed with sterile phosphate-buffered saline (PBS, Sigma), trypsinised for 2–3′ at room temperature and centrifuged at 1000 × g for 5′. Pelleted cells were resuspended in 500μL of plain NDiff227 media supplemented with DAPI. The mCherry positive fraction was sorted from DAPI negative using the BD Influx high-speed 16-parameter fluorescence activated cell sorter.

#### qPCR

For quantitative PCR, the total RNA, extracted from cells using the PureLink RNA Mini Kit (Invitrogen), was retrotranscribed (Thermo Fischer, RevertAid Reverse Transcriptase) and the cDNA used as template for each qPCR reaction in a 15μL reaction volume. iTaq Universal SYBR Green Supermix was used with the Qiagen Rotor-Gene System. To eliminate the contamination from genomic DNA, the RNeasy Plus Mini Kit (Qiagen) was used to purify the total RNA used for the RNA Sequencing. Oligos are reported in Table S8.

#### QuantSeq 3′ RNA sequencing library preparation

Preparation of libraries was performed with a total of 100ng of RNA from each sample using QuantSeq 3'mRNA-Seq Library prep kit (Lexogen, Vienna, Austria) according to manufacturer's instructions. Total RNA was quantified using the Qubit 2.0 fluorimetric Assay (Thermo Fisher Scientific). Libraries were prepared from 100ng of total RNA using the QuantSeq 3' mRNA-Seq Library Prep Kit FWD for Illumina (Lexogen GmbH). Quality of libraries was assessed by using screen tape High sensitivity DNA D1000 (Agilent Technologies). Libraries were sequenced on a NovaSeq 6000 sequencing system using an S1, 100 cycles flow cell (Illumina Inc.). Amplified fragmented cDNA of 300 bp in size were sequenced in single-end mode with a read length of 100 bp.

Illumina novaSeq base call (BCL) files are converted in fastq file through bcl2fastq.

#### QuantSeq 3′ RNA sequencing data processing and analysis

For analysis, sequence reads were trimmed using bbduk software (bbmap suite 37.31) to remove adapter sequences, poly-A tails and low-quality end bases (regions with average quality below 6). Alignment was performed with STAR 2.6.0a3 (Dobin et al., 2013) on mm10 reference assembly obtained from cellRanger website (https://support.10xgenomics.com/single-cell-gene-expression/software/release-notes/build#mm10_3.0.0; Ensembl assembly release 93). Expression levels of genes were determined with htseq-count (Anders et al., 2015) using Gencode/Ensembl gene model. We have filtered out all genes having <1 cpm in less than n_min samples and Perc MM reads >20% simultaneously. Differential expression analysis was performed using edgeR (Robinson et al., 2010), a statistical package based on generalized linear models, suitable for multifactorial experiments. The threshold for statistical significance chosen was False Discovery Rate (FDR) < 0.05 (GSE148879). The lists of differentially expressed genes (DEGs), for each comparison, with a threshold of logFC >2 for the induced and logFC <−2 for the inhibited transcripts (Table S2. Differentially expressed genes, C1M vs C1, related to Figures 3 and S5–S8, Table S3. Differentially expressed genes, C1H vs C1, related to Figures 3 and S5–S8, Table S4. Differentially expressed genes, C1MV vs C1T, related to Figure 4, Table S5. Differentially expressed genes, C1MDT vs C1T, related to Figure 4, Table S6. Differentially expressed genes, C1HV vs C1T, related to Figure 4, Table S7. Differentially expressed genes, C1HDT vs C1T, related to Figure 4) were used for the Functional Annotation analysis.

#### Weighted gene correlation network analysis (WGCNA)

Quant-seq 3′ mRNA data of 32 samples was used to construct a gene co-expression network by applying Weighted Gene Correlation Network Analysis (WGCNA)(Langfelder and Horvath, 2008) from the WGCNA package in the R statistical environment version 3.6. Briefly, we first computed the Pearson correlation coefficient among all pairs of expressed genes and then an appropriate value of the soft-thresholding power (β = 6) giving a scale-free topology fitting index (R^2^) ≥ 0.85 was selected to build the weighted adjacency matrix. The weighted adjacency matrix was further transformed into a topological overlap matrix (TOM) (Yip and Horvath, 2007) and the resulting dissimilarity matrix used for hierarchical clustering. Gene modules were finally identified by cutting the hierarchical dendrogram with the dynamic tree cut algorithm from dynamicTreeCut package in R (Langfelder et al., 2008) statistical environment with standard parameters, except for cutHeight we set equal to 0.25 and deepSplit we set equal to 1. The value of deepSplit parameter was selected after performing a cluster stability analysis. Briefly, for each possible value of deepSplit parameter (i.e., 0, 1, 2, 3 or 4), modules were identified for both the full dataset and 50 resampled datasets. Then, the clustering solution obtained for the full dataset was compared with each resampled solution by mean of Adjusted Rand Index (ARI) (Hubert and Arabie, 1985). The solution giving the highest average ARI was used for the clustering analysis as described above. Finally, to identify which clusters were correlated with β-catenin expression doses or differentiation time we correlated the first principal component of each gene module (i.e., the eigenmodule) with the traits of interest. The eigenmodule can be considered as a “signature” of the module gene expression. Modules correlated with the traits with a p-value < 0.01 were considered statistically significant and used for further analyses.

#### Functional annotation analysis

Differentially expressed genes (either logFC >2 or logFC <−2) and module “hubs” having high module membership (also known as |KME| > 0.8) within the module were analysed for the enrichment in GO Biological Processes (Ashburner et al., 2000) and KEGG Pathways (Kanehisa and Goto, 2000) via the clusterProfiler package in R statistical environment (Yu et al., 2012). The threshold for statistical significance was FDR <0.05, the top-ten BPs were represented as−log10 (FDR; Figures S7–S10).

### Quantification and statistical analysis

Differences between samples were analysed by two-tailed unpaired t-test and one-way ANOVA with Bonferroni’s multiple comparison test using GraphPad. A p-value lower than 0.05 was considered statistically significant.

Clustergram over heatmaps were generated using the clustergram function in Matlab that applies the Euclidean distance metric and average linkage. The data have been standardized across all samples for each gene and have 0 as mean and 1 as standard deviation.

## Data Availability

• RNAseq raw data and analyses have been deposited on GEO: GSE148879. The GEO accession number is also listed in the key resources table. • This paper did not report any original code. • Additional information about this study is available from the lead contacts upon request.

## Supplemental information

Document S1. Figures S1–S10

Table S1. Identified gene modules correlating with time or β-catenin doses, related to Figures 3 and S5–S8 List of identified module genes correlating with time (Figure S3) or β-catenin doses (Figure 3) before and after the filtering for the |kME| ≥ 0.8; GO and pathway analysis.

Table S2. Differentially expressed genes, C1M vs C1, related to Figures 3 and S5–S8 List of differentially expressed genes before and after the filtering for the FDR (< 0.05) and log fold change (−2 > log > 2); GO and pathway analysis in pluripotent C1M vs C1 ESCs.

Table S3. Differentially expressed genes, C1H vs C1, related to Figures 3 and S5–S8 List of differentially expressed genes before and after the filtering for the FDR (< 0.05) and log fold change (−2 > log > 2); GO and pathway analysis in pluripotent C1H vs C1 ESCs.

Table S4. Differentially expressed genes, C1MV vs C1T, related to Figure 4 List of differentially expressed genes before and after the filtering for the FDR (< 0.05) and log fold change (−2 > log > 2); GO and pathway analysis in differentiated C1MV vs C1T ESCs.

Table S5. Differentially expressed genes, C1MDT vs C1T, related to Figure 4 List of differentially expressed genes before and after the filtering for the FDR (< 0.05) and log fold change (−2 > log > 2); GO and pathway analysis in differentiated C1MDT vs C1T ESCs.

Table S6. Differentially expressed genes, C1HV vs C1T, related to Figure 4 List of differentially expressed genes before and after the filtering for the FDR (< 0.05) and log fold change (−2 > log > 2); GO and pathway analysis in differentiated C1HV vs C1T ESCs.

Table S7. Differentially expressed genes, C1HDT vs C1T, related to Figure 4 List of differentially expressed genes before and after the filtering for the FDR (< 0.05) and log fold change (−2 > log > 2); GO and pathway analysis in differentiated C1HDT vs C1T ESCs.

Table S8. Oligonucleotides, related to Figures 1, 2, and S1–S5 List of oligonucleotides used in this study.
