# Supplementary material for: β-catenin perturbations control differentiation programs in mouse embryonic stem cells
Source: iScience. 2022 Jan 10;25(2):103756. doi: 10.1016/j.isci.2022.103756 (PMC8804270; doi:10.1016/j.isci.2022.103756)
Supplement: Document S1. Figures S1–S10 [file mmc1.pdf]

iScience, Volume *25*

Supplemental information

β-catenin perturbations control differentiation programs in mouse embryonic stem cells

Elisa Pedone, Mario Failli, Gennaro Gambardella, Rossella De Cegli, Antonella La Regina, Diego di Bernardo, and Lucia Marucci

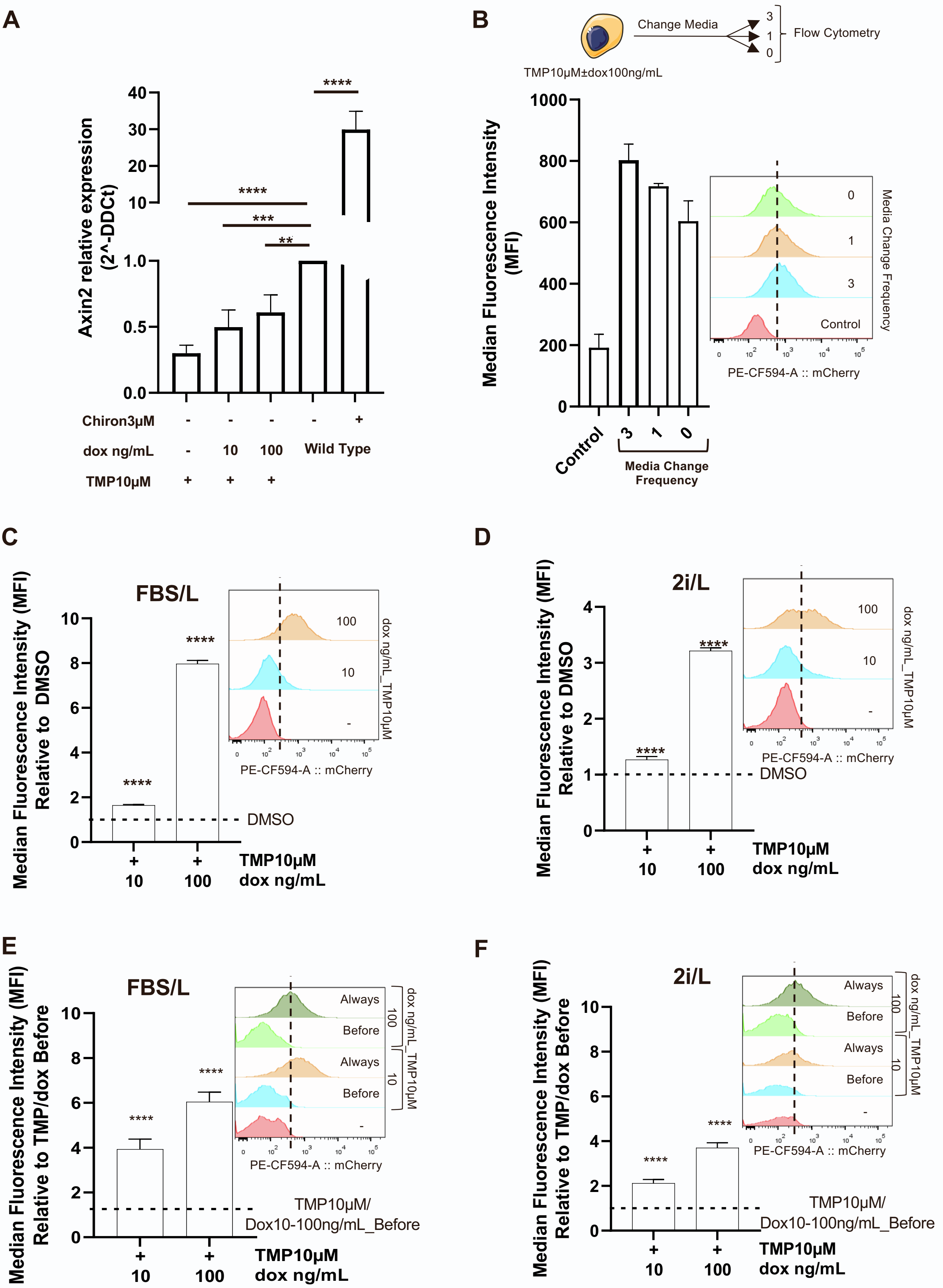
A
Axin2 relative expression (2^-DDCt)
****
***
**
****
Chiron3μM
dox ng/mL
TMP10μM
Wild Type
B
Change Media
Flow Cytometry
TMP10μM±dox100ng/mL
Median Fluorescence Intensity (MFI)
Control
Media Change Frequency
PE-CF594-A :: mCherry
C
FBS/L
Median Fluorescence Intensity (MFI) Relative to DMSO
DMSO
TMP10μM
dox ng/mL
dox ng/mL_TMP10μM
D
2i/L
E
Median Fluorescence Intensity (MFI) Relative to TMP/dox Before
Always
Before
TMP10μM/
Dox10-100ng/mL_Before
F

Figure S1

Figure S1. Characterization of β-catenin overexpressing C1 ESCs and in EpiLC *in vitro* derivation (Related to Figure 1).

A Axin2 expression in C1 ESCs treated for 48 hrs with dox10-100ng/mL and/or TMP10μM, and wild type ESCs stimulated or not with Chiron3μM. **(B)** mCherry Median Fluorescence Intensity (MFI) of C1 ESCs cultured for 4 days in FBS/L supplemented with TMP10μM and dox10ng/mL. Media was changed and refreshed after 24 (3) and 48 (1) hrs in culture. Control cells were kept in the same media for the entire duration of the experiment without changing the media (0). **C-F** mCherry Median Fluorescence Intensity (MFI) of FBS/L **(C, E)** and 2i/L **(D, F)** C1 ESCs grown in pluripotent **(C, D)** and differentiating **(E, F)** culture conditions. Both pluripotent and differentiation media were supplemented with DMSO (i.e., time zero negative control or TMP10μM_dox10-100ng/mL_Before) or TMP10μM and dox10-100ng/mL (i.e., TMP10μM_dox10-100ng/mL_Always). Flow cytometry histograms are shown as inset.

Data are represented as fold-change with respect to unstimulated wild type ESCs (A), DMSO-treated cells (C, D), or TMP10μM_dox10-100ng/mL_Before treatments (E, F), as indicated by the dashed lines.

Data are means±SEM (n=3, A, C-F; n=2, B, biological replicates). p-values from two-tailed unpaired t test (A, C-F) computed over the wild type (A) or DMSO-treated C1 (C-F) ESCs are shown, *p<0.05, **p<0.01, ***p<0.001, ****p<0.0001.

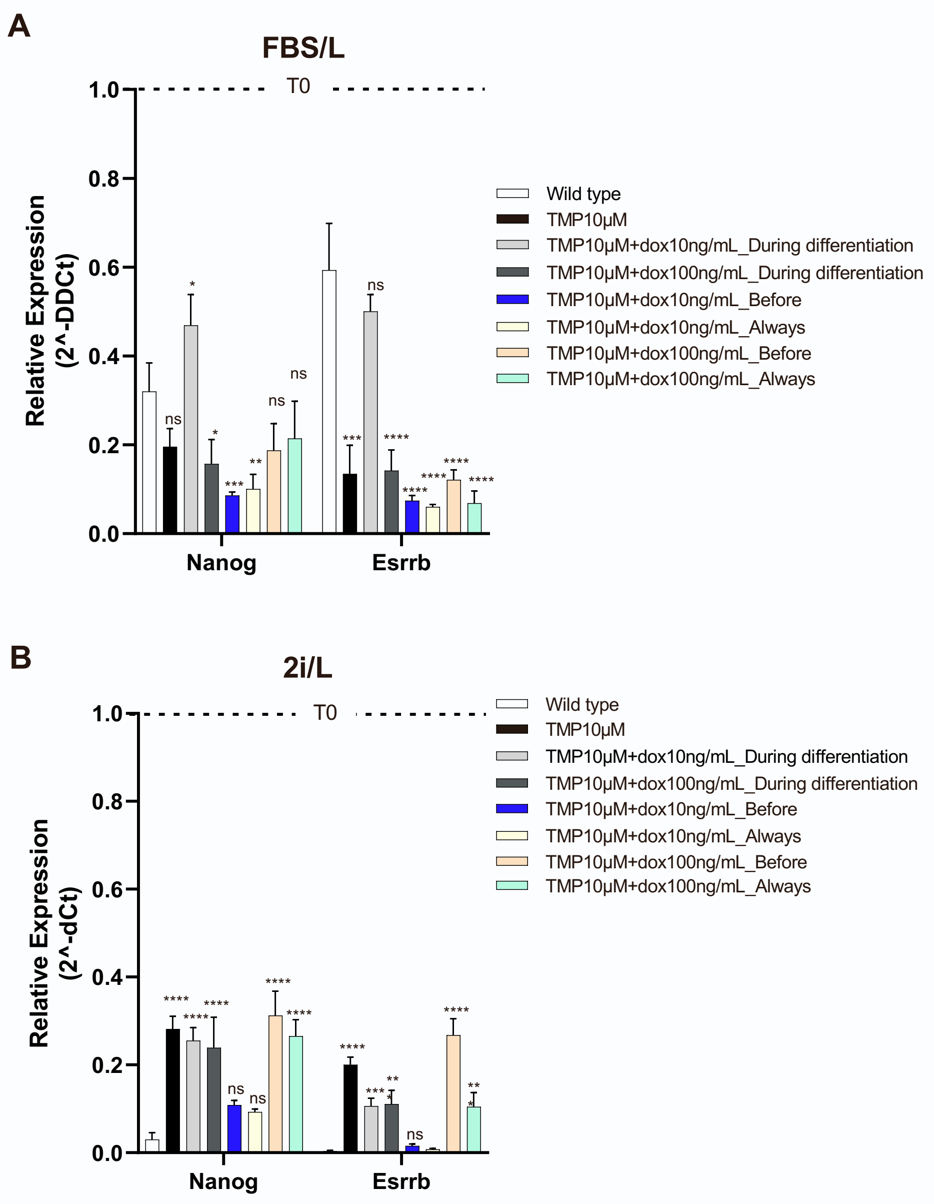
A
FBS/L
T0
Relative Expression (2^-DDCt)
Nanog
Esrrb
Wild type
TMP10μM
TMP10μM+dox10ng/mL_During differentiation
TMP10μM+dox100ng/mL_During differentiation
TMP10μM+dox10ng/mL_Before
TMP10μM+dox10ng/mL_Always
TMP10μM+dox100ng/mL_Before
TMP10μM+dox100ng/mL_Always
B
2i/L
T0
Relative Expression (2^-dCt)
Nanog
Esrrb

Figure S2

Figure S2. Pluripotency gene expression in EpiLC *in vitro* derivation (Related to Figure 1).

A, B Nanog and Esrrb expression in C1 ESCs cultured in FBS/L **(A)** or 2i/L **(B)** and differentiated for 4 days in NDiff227+ActivinA/FGF2 and different combination of DMSO, doxy and TMP. Data are represented as fold-change with respect to the corresponding pluripotent condition (i.e., time zero before differentiation (T0)), indicated with a dashed line. Data are means±SEM (n=3 biological replicates). p-values from one-way ANOVA with Bonferroni's multiple comparison test computed over the wild type ESCs are shown, *p<0.05, **p<0.01, ***p<0.001, ****p<0.0001.

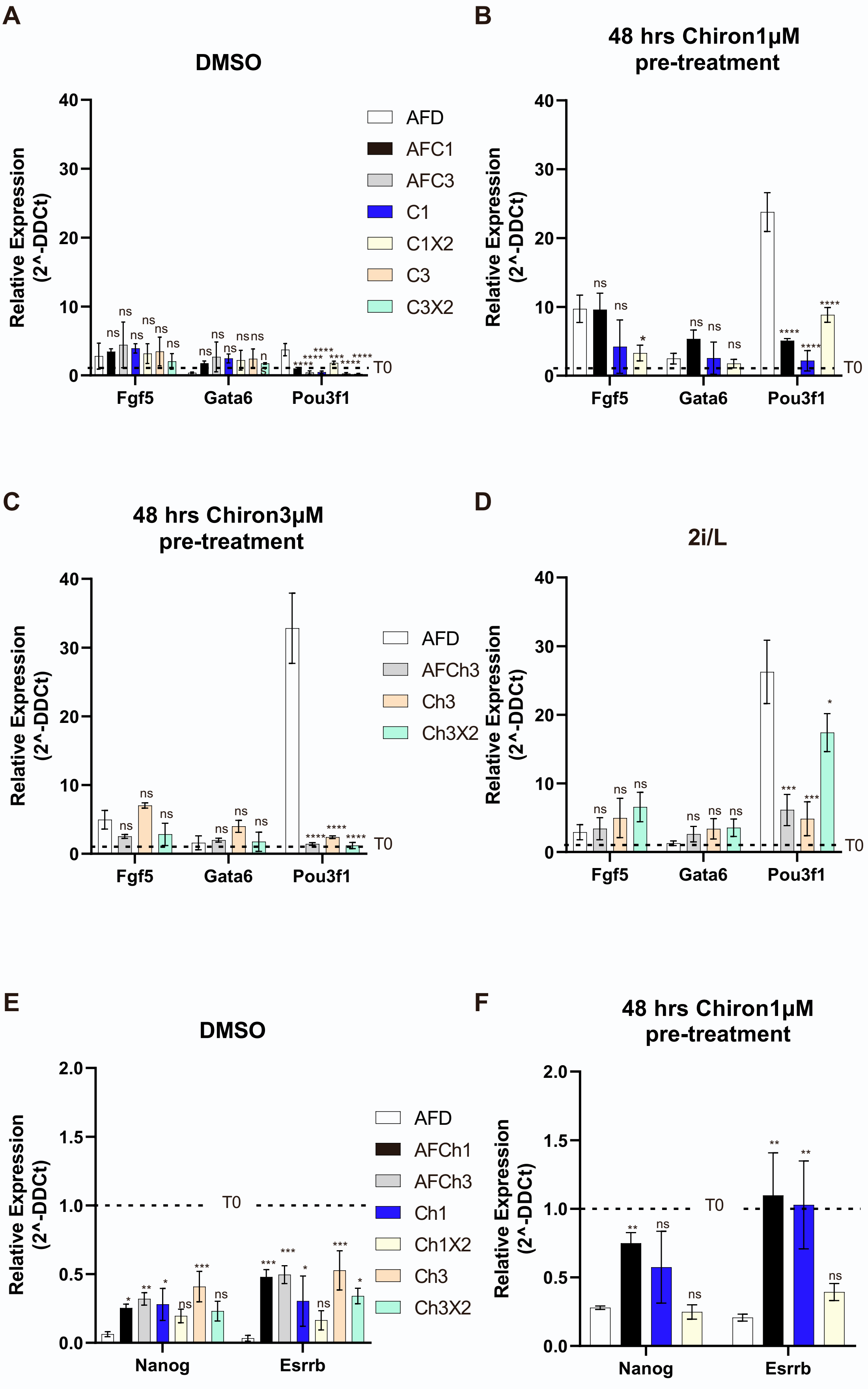
A
DMSO
Relative Expression
(2^-DDCt)
AFD
AFC1
AFC3
C1
C1X2
C3
C3X2
T0
Fgf5
Gata6
Pou3f1
B
48 hrs Chiron1μM
pre-treatment
Relative Expression
(2^-DDCt)
T0
Fgf5
Gata6
Pou3f1
C
48 hrs Chiron3μM
pre-treatment
Relative Expression
(2^-DDCt)
AFD
AFCh3
Ch3
Ch3X2
T0
Fgf5
Gata6
Pou3f1
D
2i/L
Relative Expression
(2^-DDCt)
T0
Fgf5
Gata6
Pou3f1
E
DMSO
Relative Expression
(2^-DDCt)
T0
AFD
AFCh1
AFCh3
Ch1
Ch1X2
Ch3
Ch3X2
Nanog
Esrrb
F
48 hrs Chiron1μM
pre-treatment
Relative Expression
(2^-DDCt)
T0
Nanog
Esrrb

Figure S3

Figure S3. Characterization of wild type ESCs exposed to chemical perturbation of the Wnt/β-catenin pathway (Related to Figure 2).

A-D Fgf5, Gata6 and Pou3f1 expression in DMSO **(A)**, Ch1μM **(B)**, Ch3μM **(C)** and 2i/L **(D)** pre-cultured wild type ESCs differentiated for 4 days in NDiff227 and the combination of drugs indicated in Figure 2. **E, F** Nanog and Esrrb expression in DMSO **(E)** and Ch1μM **(F)** pre-cultured wild type ESCs differentiated for 4 days in NDiff227 and the combination of drugs indicated in Figure 2.

Data are represented as fold-change with respect to the corresponding pluripotent condition (i.e., time zero before differentiation (T0), indicated with a dashed line). Data are means±SEM (n=3 biological replicates). p-values from one-way ANOVA with Bonferroni's multiple comparison test computed over the standard differentiation protocol based on Activin A and FGF2 (i.e. AFD) are shown, *p<0.05, **p<0.01, ***p<0.001, ****p<0.0001.

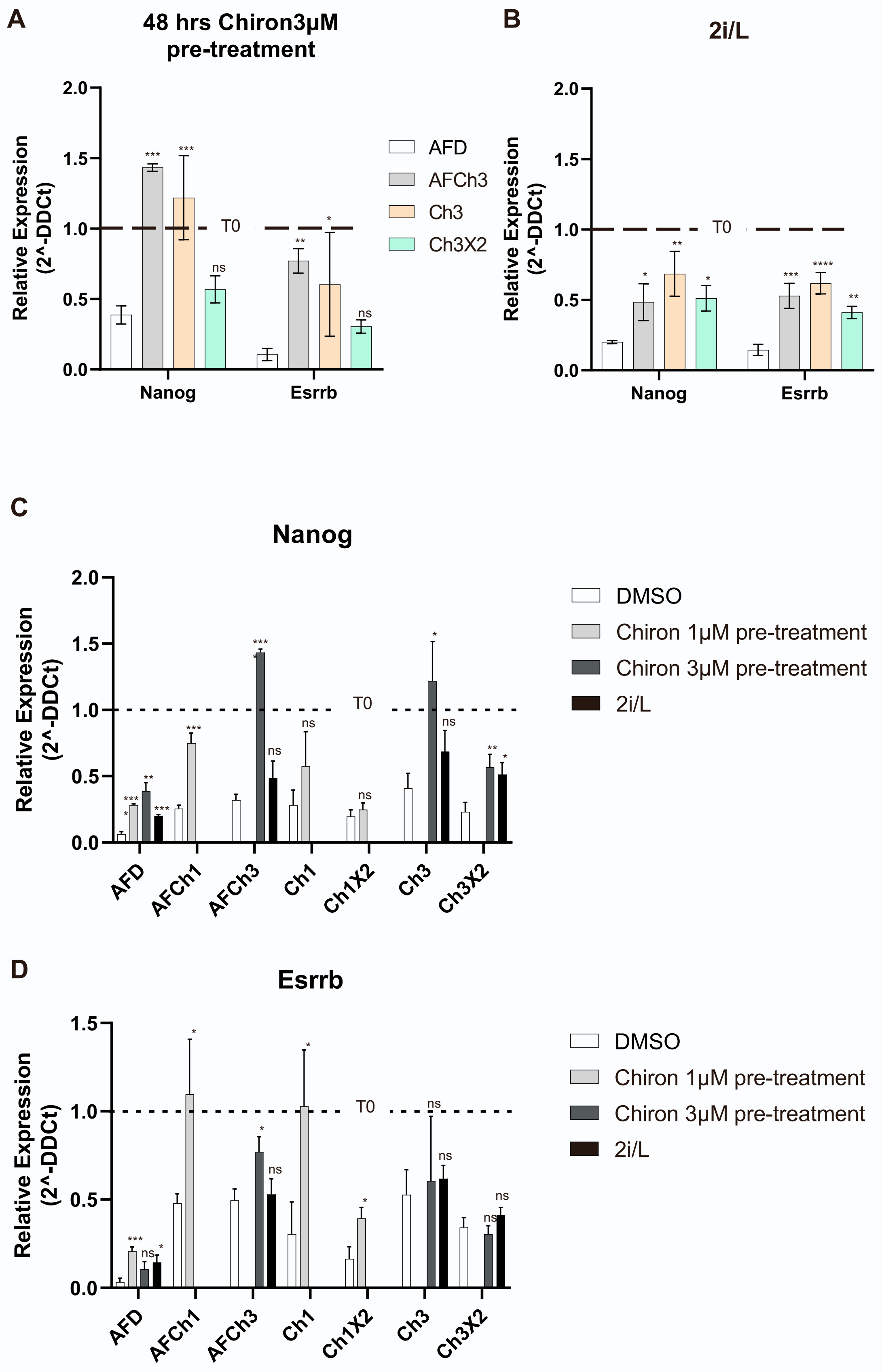

A
48 hrs Chiron3μM pre-treatment
Relative Expression (2^-DDCt)
T0
AFD
AFCh3
Ch3
Ch3X2
Nanog
Esrrb
B
2i/L
C
Nanog
DMSO
Chiron 1μM pre-treatment
Chiron 3μM pre-treatment
2i/L
AFD
AFCh1
AFCh3
Ch1
Ch1X2
Ch3
Ch3X2
D
Esrrb

Figure S4

Figure S4. Further characterization of wild type ESCs exposed to chemical perturbation of the Wnt/β-catenin pathway (Related to Figure 2).

A, B Nanog and Esrrb expression in Ch3μM **(A)** and 2i/L **(B)** pre-cultured wild type ESCs differentiated for 4 days in NDiff227 and the combination of drugs indicated in Figure 2. **C, D** Nanog **(C)** and Esrrb **(D)** expression in DMSO, Ch1μM, Ch3μM and 2i/L pre-cultured wild type ESCs differentiated for 4 days in NDiff227 and the combination of drugs indicated in Figure 2.

Data are represented as fold-change with respect to the corresponding pluripotent condition (i.e., time zero before differentiation (T0), indicated with a dashed line). Data are means±SEM (n=3 biological replicates). p-values from one-way ANOVA with Bonferroni's multiple comparison test (A-B) and two-tailed unpaired t test (C, D) computed over the standard differentiation protocol based on ActivinA and FGF2 (i.e. AFD, A, B) or DMSO (C, D) are shown, *p<0.05, **p<0.01, ***p<0.001, ****p<0.0001.

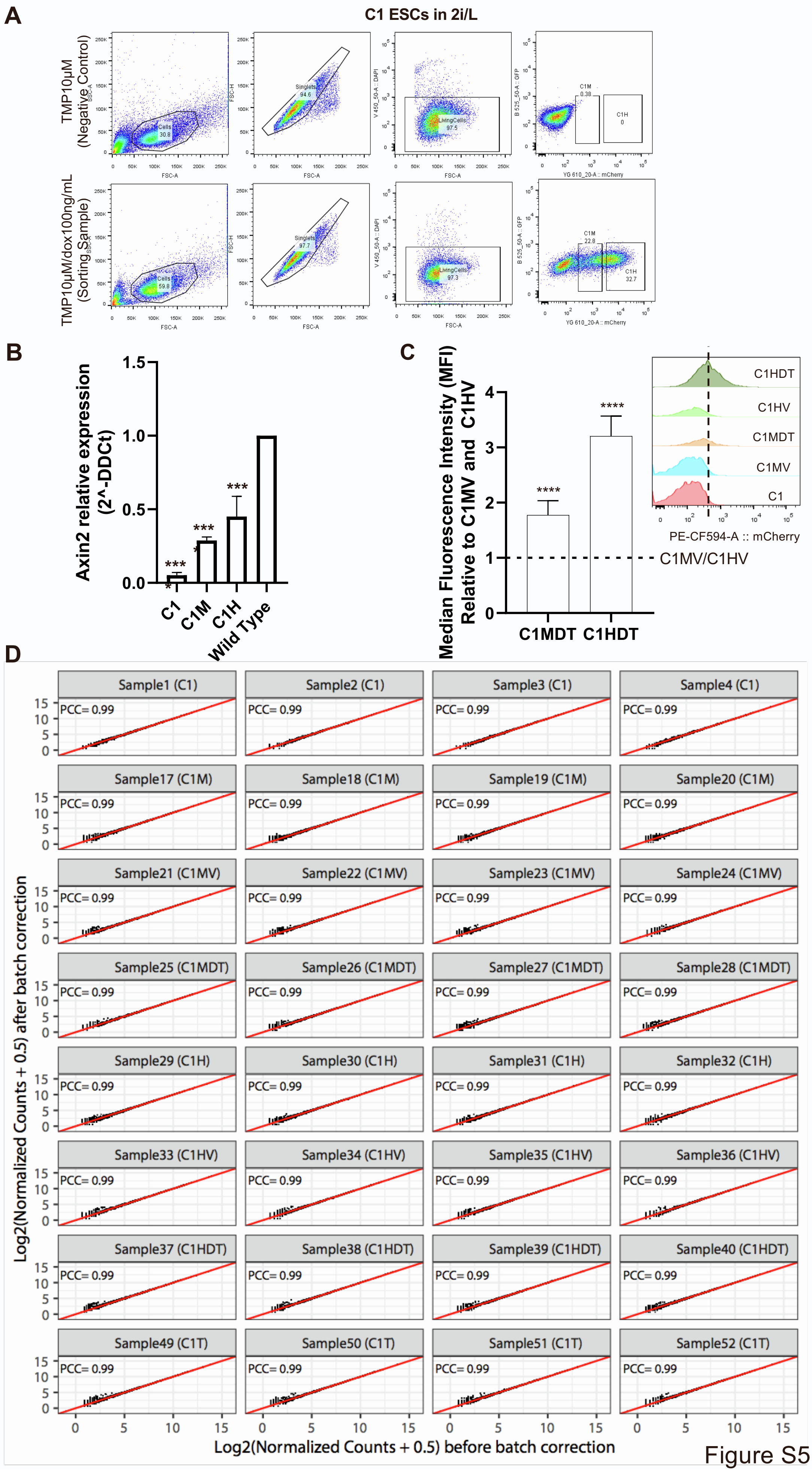
A
C1 ESCs in 2i/L
TMP10μM
(Negative Control)
TMP10μM/dox100ng/mL
(Sorting Sample)
Cells
30.8
59.8
Singlets
94.6
97.7
LivingCells
97.5
97.3
C1M
0.38
22.8
C1H
0
32.7
FSC-A
SSC-A
FSC-H
V 450_50-A :: DAPI
B 525_50-A :: GFP
YG 610_20-A :: mCherry
B
Axin2 relative expression
(2^-DDCt)
1.5
1.0
0.5
0.0
***
***
***
C1
C1M
C1H
Wild Type
C
Median Fluorescence Intensity (MFI)
Relative to C1MV and C1HV
4
3
2
1
0
****
****
C1MV/C1HV
C1MDT
C1HDT
C1HDT
C1HV
C1MDT
C1MV
C1
PE-CF594-A :: mCherry
D
Sample1 (C1)
Sample2 (C1)
Sample3 (C1)
Sample4 (C1)
Sample17 (C1M)
Sample18 (C1M)
Sample19 (C1M)
Sample20 (C1M)
Sample21 (C1MV)
Sample22 (C1MV)
Sample23 (C1MV)
Sample24 (C1MV)
Sample25 (C1MDT)
Sample26 (C1MDT)
Sample27 (C1MDT)
Sample28 (C1MDT)
Sample29 (C1H)
Sample30 (C1H)
Sample31 (C1H)
Sample32 (C1H)
Sample33 (C1HV)
Sample34 (C1HV)
Sample35 (C1HV)
Sample36 (C1HV)
Sample37 (C1HDT)
Sample38 (C1HDT)
Sample39 (C1HDT)
Sample40 (C1HDT)
Sample49 (C1T)
Sample50 (C1T)
Sample51 (C1T)
Sample52 (C1T)
PCC= 0.99
Log2(Normalized Counts + 0.5) after batch correction
Log2(Normalized Counts + 0.5) before batch correction
0
5
10
15

Figure S5

Figure S5. FACS gating strategy and scatter plots of gene expression (Related to Figure 3).

A Gating strategy used to sort C1M and C1H ESCs, following 48 hrs treatment with TMP10µM and dox100ng/mL. C1 ESCs treated with TMP10µM were used as negative control. **B** Axin2 expression in C1, C1M, C1H and wild type ESCs. **C** mCherry Median Fluorescence Intensity (MFI) of pluripotent C1MDT and C1HDT ESCs under differentiating culture conditions. Flow cytometry histogram is shown as inset. **D** Scatter plots of gene expression profiles, across all the 32 samples, before (x-axis) and after (y-axis) the batch correction. The Pearson correlation coefficient (PCC) is reported for each comparison.

Data are represented as fold-change with respect to wild type (B) or C1MV and C1HV (C, indicated with a dashed line) ESCs. p-values from two-tailed unpaired t test computed over the wild type (B) or C1MV and C1HV (C) ESCs are shown, *$p<0.05$, **$p<0.01$, ***$p<0.001$, ****$p<0.0001$.

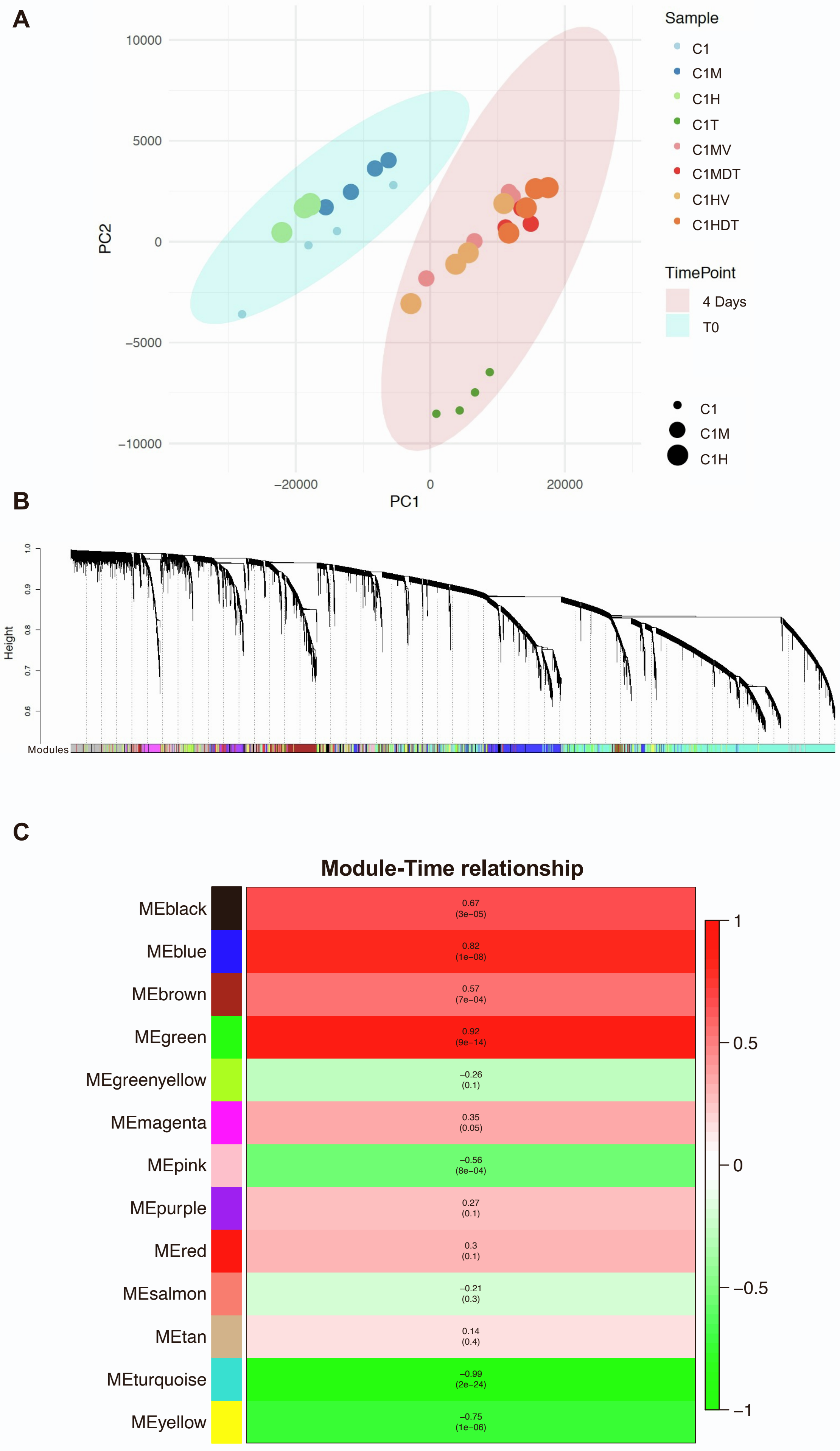
A
PC1
PC2
10000
5000
0
−5000
−10000
−20000
0
20000
Sample
C1
C1M
C1H
C1T
C1MV
C1MDT
C1HV
C1HDT
TimePoint
4 Days
T0
C1
C1M
C1H
B
Height
1.0
0.9
0.8
0.7
0.6
Modules
C
Module-Time relationship
MEblack
MEblue
MEbrown
MEgreen
MEgreenyellow
MEmagenta
MEpink
MEpurple
MEred
MEsalmon
MEtan
MEturquoise
MEyellow
0.67 (3e−05)
0.82 (1e−08)
0.57 (7e−04)
0.92 (9e−14)
−0.26 (0.1)
0.35 (0.05)
−0.56 (8e−04)
0.27 (0.1)
0.3 (0.1)
−0.21 (0.3)
0.14 (0.4)
−0.99 (2e−24)
−0.75 (1e−06)
1
0.5
0
−0.5
−1

Figure S6

Figure S6. Sequencing samples PCA, and WGCNA of the genes correlating with time (Related to Figure 3).

A Principal Component Analysis (PCA) of all samples; the average of replica is shown.

B Clustering dendrogram of genes, with dissimilarity based on topological overlap, together with the assigned module colours**;** grey genes are unassigned to any module**.**

C Eigenmodules correlating with time; the Pearson correlation coefficient (*r*) and relative p-value are shown.

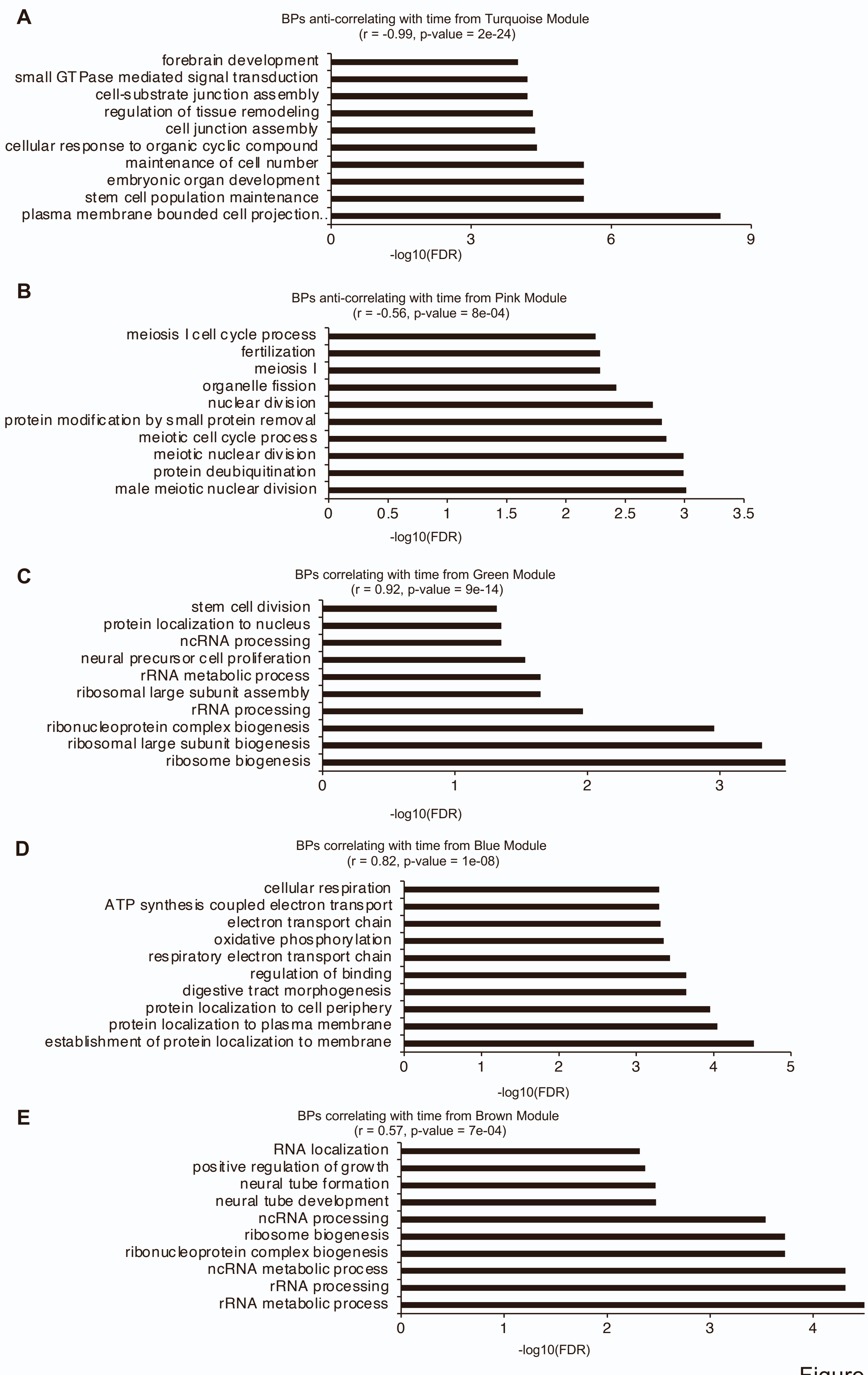
A
BPs anti-correlating with time from Turquoise Module
(r = -0.99, p-value = 2e-24)
forebrain development
small GTPase mediated signal transduction
cell-substrate junction assembly
regulation of tissue remodeling
cell junction assembly
cellular response to organic cyclic compound
maintenance of cell number
embryonic organ development
stem cell population maintenance
plasma membrane bounded cell projection..
0
3
6
9
-log10(FDR)
B
BPs anti-correlating with time from Pink Module
(r = -0.56, p-value = 8e-04)
meiosis I cell cycle process
fertilization
meiosis I
organelle fission
nuclear division
protein modification by small protein removal
meiotic cell cycle process
meiotic nuclear division
protein deubiquitination
male meiotic nuclear division
0
0.5
1
1.5
2
2.5
3
3.5
-log10(FDR)
C
BPs correlating with time from Green Module
(r = 0.92, p-value = 9e-14)
stem cell division
protein localization to nucleus
ncRNA processing
neural precursor cell proliferation
rRNA metabolic process
ribosomal large subunit assembly
rRNA processing
ribonucleoprotein complex biogenesis
ribosomal large subunit biogenesis
ribosome biogenesis
0
1
2
3
-log10(FDR)
D
BPs correlating with time from Blue Module
(r = 0.82, p-value = 1e-08)
cellular respiration
ATP synthesis coupled electron transport
electron transport chain
oxidative phosphorylation
respiratory electron transport chain
regulation of binding
digestive tract morphogenesis
protein localization to cell periphery
protein localization to plasma membrane
establishment of protein localization to membrane
0
1
2
3
4
5
-log10(FDR)
E
BPs correlating with time from Brown Module
(r = 0.57, p-value = 7e-04)
RNA localization
positive regulation of growth
neural tube formation
neural tube development
ncRNA processing
ribosome biogenesis
ribonucleoprotein complex biogenesis
ncRNA metabolic process
rRNA processing
rRNA metabolic process
0
1
2
3
4
-log10(FDR)

Figure S7

Figure S7. WGCNA of the genes correlating with time (Related to Figure 3).

A-E Bar-chart of the top-ten enriched biological processes (BP) with FDR < 0.05 from genes belonging to the turquoise **(A)**, Pink **(B),** Green **(C)**, Blue **(D)** and Brown **(E)**, WGCNA modules.

A

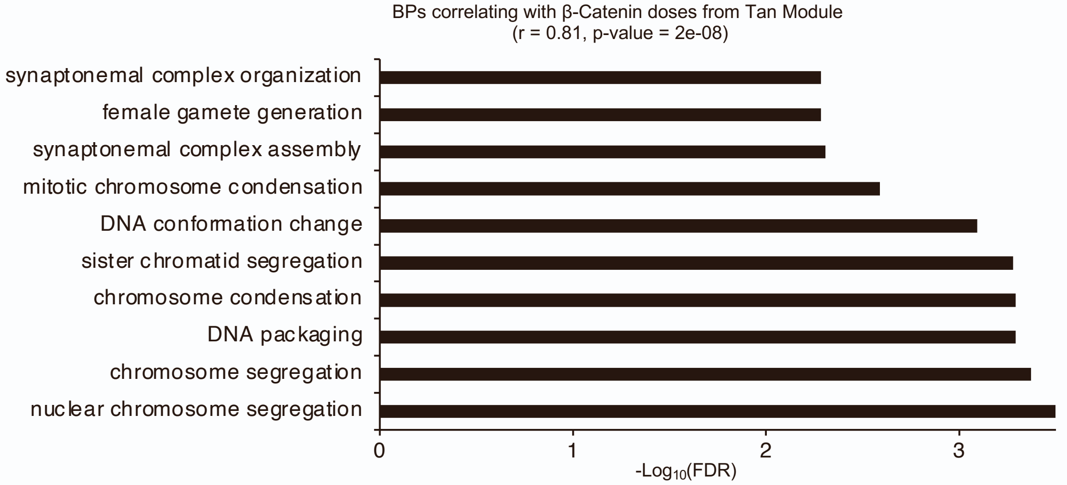
BPs correlating with β-Catenin doses from Tan Module
(r = 0.81, p-value = 2e-08)
synaptonemal complex organization
female gamete generation
synaptonemal complex assembly
mitotic chromosome condensation
DNA conformation change
sister chromatid segregation
chromosome condensation
DNA packaging
chromosome segregation
nuclear chromosome segregation
0
1
2
3
-Log10(FDR)

B

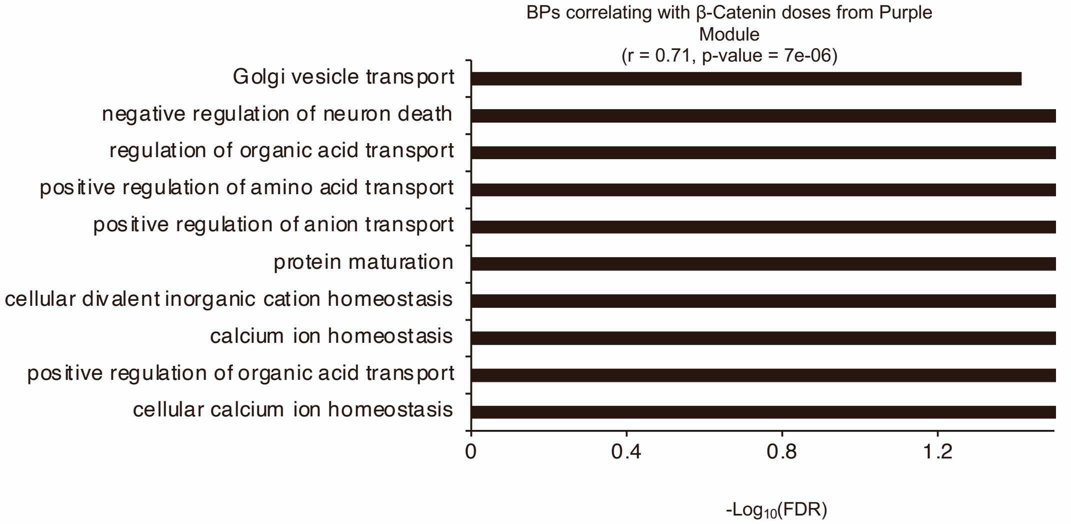
BPs correlating with β-Catenin doses from Purple Module
(r = 0.71, p-value = 7e-06)
Golgi vesicle transport
negative regulation of neuron death
regulation of organic acid transport
positive regulation of amino acid transport
positive regulation of anion transport
protein maturation
cellular divalent inorganic cation homeostasis
calcium ion homeostasis
positive regulation of organic acid transport
cellular calcium ion homeostasis
0
0.4
0.8
1.2
-Log10(FDR)

Figure S8

Figure S8. Further WGCNA results (Related to Figure 3).

A, B Bar-chart of the top-ten enriched biological processes (BP) with FDR < 0.05 from genes belonging to the Tan **(A)** and Purple **(B)** WGCNA modules.

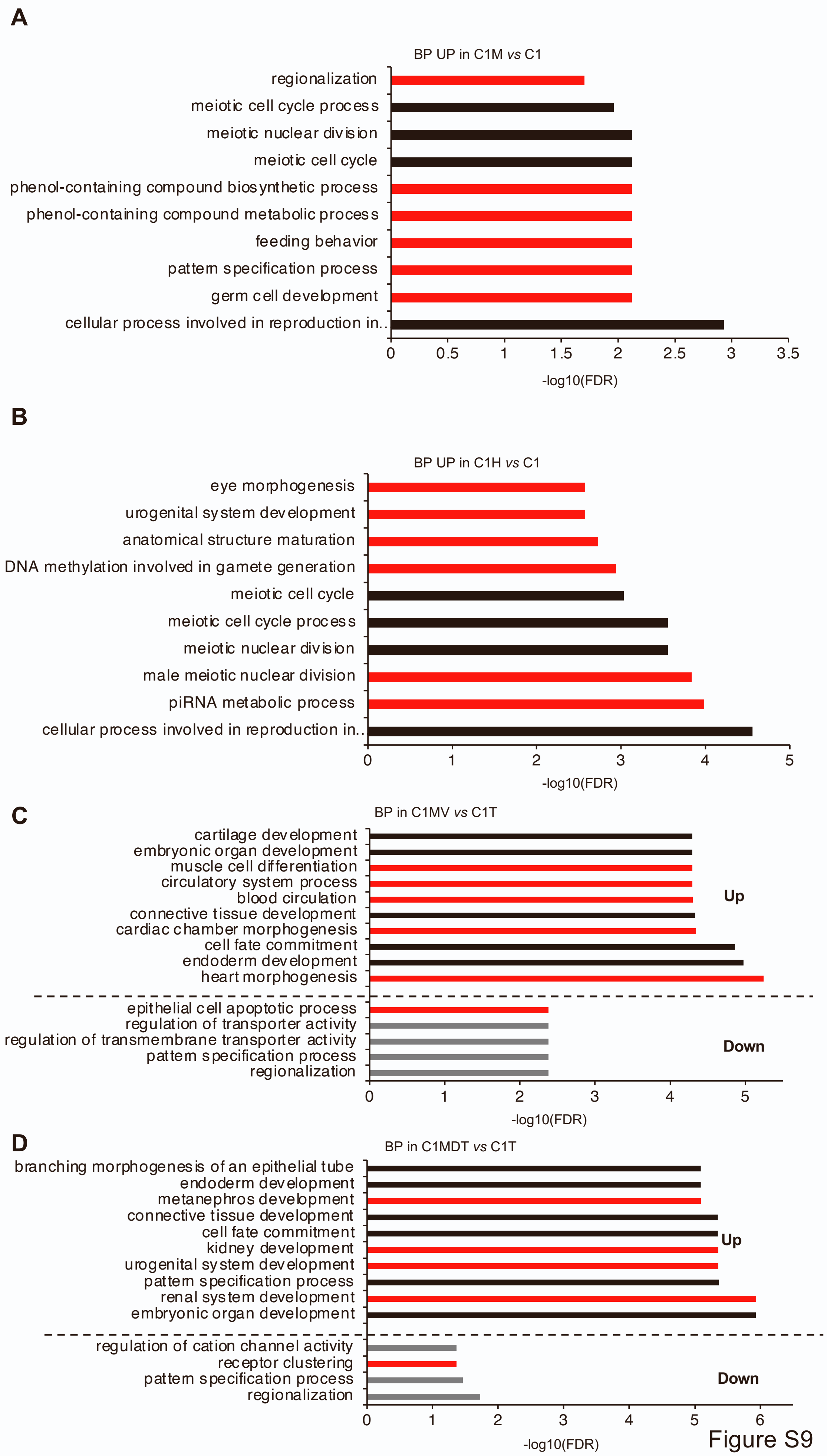
A
BP UP in C1M vs C1
regionalization
meiotic cell cycle process
meiotic nuclear division
meiotic cell cycle
phenol-containing compound biosynthetic process
phenol-containing compound metabolic process
feeding behavior
pattern specification process
germ cell development
cellular process involved in reproduction in..
0
0.5
1
1.5
2
2.5
3
3.5
-log10(FDR)
B
BP UP in C1H vs C1
eye morphogenesis
urogenital system development
anatomical structure maturation
DNA methylation involved in gamete generation
meiotic cell cycle
meiotic cell cycle process
meiotic nuclear division
male meiotic nuclear division
piRNA metabolic process
cellular process involved in reproduction in..
0
1
2
3
4
5
-log10(FDR)
C
BP in C1MV vs C1T
cartilage development
embryonic organ development
muscle cell differentiation
circulatory system process
blood circulation
connective tissue development
cardiac chamber morphogenesis
cell fate commitment
endoderm development
heart morphogenesis
Up
epithelial cell apoptotic process
regulation of transporter activity
regulation of transmembrane transporter activity
pattern specification process
regionalization
Down
0
1
2
3
4
5
-log10(FDR)
D
BP in C1MDT vs C1T
branching morphogenesis of an epithelial tube
endoderm development
metanephros development
connective tissue development
cell fate commitment
kidney development
urogenital system development
pattern specification process
renal system development
embryonic organ development
Up
regulation of cation channel activity
receptor clustering
pattern specification process
regionalization
Down
0
1
2
3
4
5
6
-log10(FDR)

Figure S9

Figure S9. Gene ontology of the differential expressed genes in pluripotent ESCs (Related to Figure 4)

A, B Bar-chart of the top-ten enriched biological processes (BP) with FDR < 0.05 from differentially expressed genes in in C1M **(A)** and C1H **(B)** compared to C1 ESCs. **C, D** Bar-chart of the top-ten enriched biological processes (BP) with FDR < 0.05 from differentially expressed genes in C1MV **(C)** and C1MDT **(D)** compared to C1T ESCs. Black and grey bars represent upregulated and downregulated BPs, respectively. In red bars, the BPs exclusively enriched in the indicated condition.

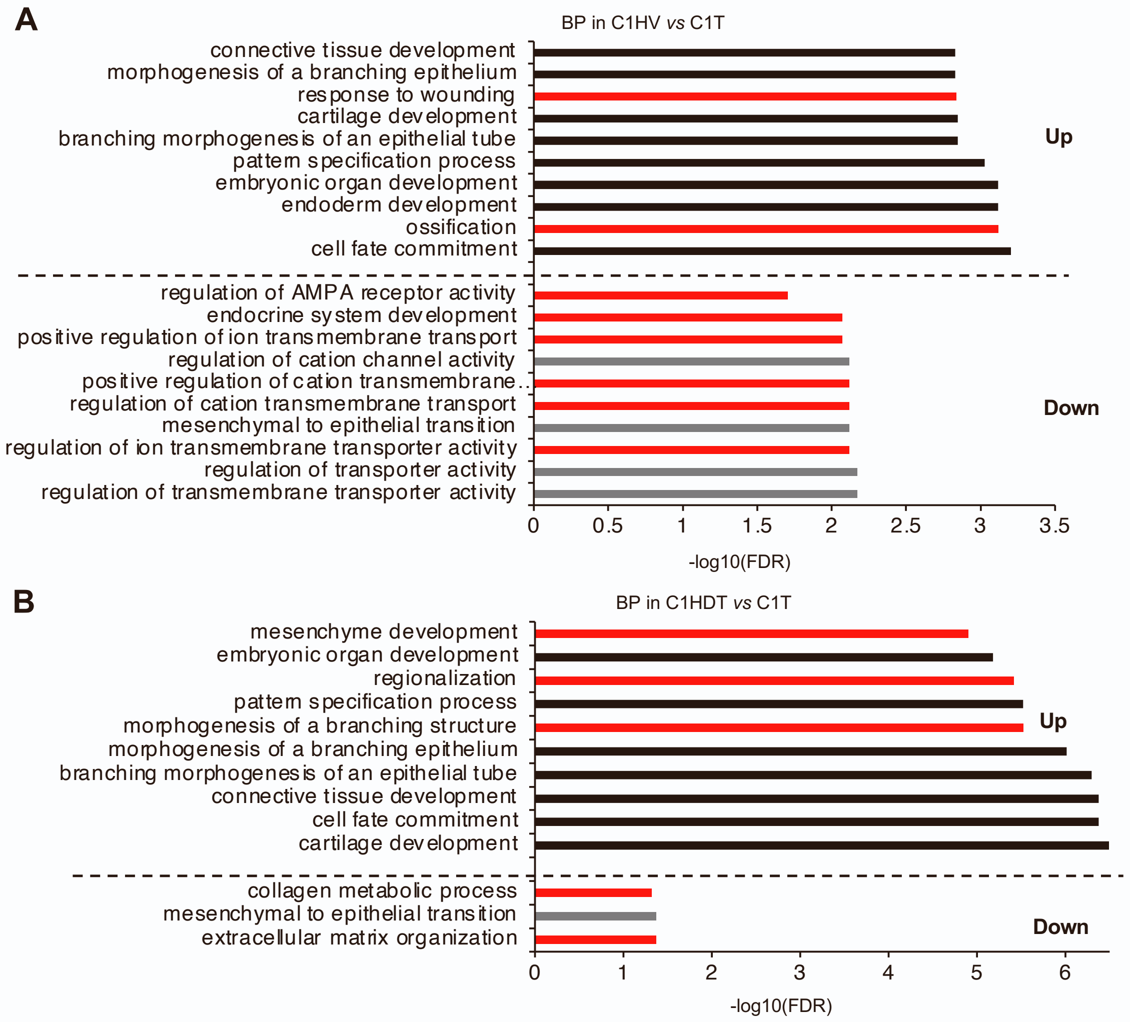
A
BP in C1HV vs C1T
connective tissue development
morphogenesis of a branching epithelium
response to wounding
cartilage development
branching morphogenesis of an epithelial tube
pattern specification process
embryonic organ development
endoderm development
ossification
cell fate commitment
Up
regulation of AMPA receptor activity
endocrine system development
positive regulation of ion transmembrane transport
regulation of cation channel activity
positive regulation of cation transmembrane…
regulation of cation transmembrane transport
mesenchymal to epithelial transition
regulation of ion transmembrane transporter activity
regulation of transporter activity
regulation of transmembrane transporter activity
Down
0
0.5
1
1.5
2
2.5
3
3.5
-log10(FDR)
B
BP in C1HDT vs C1T
mesenchyme development
embryonic organ development
regionalization
pattern specification process
morphogenesis of a branching structure
morphogenesis of a branching epithelium
branching morphogenesis of an epithelial tube
connective tissue development
cell fate commitment
cartilage development
Up
collagen metabolic process
mesenchymal to epithelial transition
extracellular matrix organization
Down
0
1
2
3
4
5
6
-log10(FDR)

Figure S10

Figure S10. Further gene ontology of the differential expressed genes in pluripotent ESCs (Related to Figure 4)

A, B Bar-chart of the top-ten enriched biological processes (BP) with FDR < 0.05 from differentially expressed genes in C1HV **(A)** and C1HDT **(B)** compared to C1T ESCs. Black and grey bars represent upregulated and downregulated BPs, respectively. In red bars, the BPs exclusively enriched in the indicated condition.
